# Structural Characteristics for the Interaction of 1-Benzyl-2-Methylbenzimidazoles as Insect Growth Regulators and Juvenile Hormone Binding Protein

**DOI:** 10.3390/insects17060657

**Published:** 2026-06-22

**Authors:** Udawaththa Kankanamge Don Sahan Suganda Gunasekara, Konatsu Inoue, Zui Fujimoto, Shuhei Henmi, Wataru Tsuchiya, Rintaro Suzuki, Keisuke Kutsuwada, Izumi Ikeda, Toshimasa Yamazaki, Takahiro Shiotsuki

**Affiliations:** 1 United Graduate School of Agricultural Sciences, Tottori University, Tottori 680-0945, Japan; d23a2103@matsu.shimane-u.ac.jp; 2 Graduate School of Natural Science and Technology, Shimane University, Matsue 690-8504, Japan; w265503a@yokohama-cu.ac.jp (K.I.); shuhei-hemmi@oat-agrio.co.jp (S.H.); 3 Research Center for Advanced Analysis, National Agriculture and Food Research Organization, Tsukuba 305-8642, Ibaraki, Japan; fujimoto.zui899@naro.go.jp (Z.F.); tsuchiya.wataru974@naro.go.jp (W.T.); suzuki.rintaro377@naro.go.jp (R.S.); kutsuwada.keisuke401@naro.go.jp (K.K.); yamazaki.toshimasa752@naro.go.jp (T.Y.); 4 Department of Life Science and Biotechnology, Faculty of Life and Environmental Science, Shimane University, Matsue 690-8504, Japan; ikeda@life.shimane-u.ac.jp

**Keywords:** juvenile hormone, insect growth regulator, juvenile hormone binding protein, molting, metamorphosis, *Bombyx mori*, X-ray crystallography

## Abstract

The authors previously reported that 2-methylbenzimidazole derivatives (MBIs) exhibit insect growth-regulating activity against the silkworm, *Bombyx mori*. However, despite their unique effects on juvenile hormone (JH)-related endocrine pathways, the precise mode of action of MBIs remained unclear. In the present study, the interactions between MBIs and the lepidopteran hemolymph JH-binding protein (JHBP), a key regulator of JH transport and activity, were investigated using multiple approaches, including in vitro binding affinity assays, X-ray crystallography, and molecular docking simulations. A series of MBIs bearing a 1-(4-alkoxybenzyl) group, which exhibited potent insect growth-regulating activity, showed high binding affinity and structural compatibility with the JH-binding pocket of JHBP. In contrast, 1-(4-alkylbenzyl) MBIs, which displayed weak or negligible insect growth-regulating activity, exhibited low affinity for JHBP. These findings suggest that the insect growth-regulating activity of MBIs is mediated through inhibition of JHBP function, likely by disrupting the precise regulation of JH concentration in the hemolymph during larval development and pupal metamorphosis.

## Simple Summary

The development of insect growth regulators (IGRs) has emerged as an environmentally sustainable strategy for pest management, driven by increasing concerns that widely used neurotoxic insecticides can adversely affect a broad range of beneficial organisms. Methylbenzimidazole derivatives constitute a promising class of IGRs, as previously reported by our group; building on these findings, we synthesized a novel series of 1-benzyl-2-methylbenzimidazole (BMBI) derivatives bearing alkyl, alkoxy, and alkylamino substituents at the 4′-position of the benzyl ring and evaluated biological and binding affinity assay. The results showed that the compounds produced distinct phenotypes, particularly compounds **4** (4′-OPr) and **2** (4′-Bu), and were therefore selected for further structural analysis with one of the target juvenile hormone-binding protein (JHBP) using X-ray crystallography and molecular modeling. Crystallographic and docking analyses reveal several notable differences of conformation and interactions between the JHBP complexes of **4** (4′-OPr) and **2** (4′-Bu). These findings offer valuable guidance for further development of effective IGRs as optimize pest control strategies.

## 1. Introduction

In recent years, the development of insect growth regulators (IGRs) has emerged as an environmentally sustainable strategy for pest management, offering high selectivity toward target species while minimizing adverse effects on non-target organisms and mitigating the development of insecticide resistance [1,2,3,4]. IGRs are compounds that interfere with the growth, development and metamorphosis of insects, thereby helping to control pest populations [5]. The Insecticide Resistance Action Committee (IRAC) classifies IGRs into several groups including juvenile hormone receptor modulators (Group 7), chitin synthesis inhibitors (Groups 15 and 16), molting disruptors (Group 17), and ecdysone receptor agonists (Group 18) (IRAC 2024) [6,7,8,9,10]. Ecdysone receptor agonists, such as tebufenozide and methoxyfenozide, bind to the ecdysone receptor, inducing hyperactivation of ecdysteroid signaling and prematurely triggering cuticle synthesis through the activation of epidermal cells. This disrupts post-apolysis processes and inhibits proper ecdysis, ultimately resulting in developmental failure. The mechanism of action of molting disruptors such as cyromazine is not completely comprehended. Tebufenozide and methoxyfenozide are examples of ecdysone receptor agonists that bind to the ecdysone receptor protein, causing hyperecdysteroid activity that forces early cuticle production through epidermal cell activation [11]. Some IGRs exert their biological effects by disrupting endocrine signaling pathways that govern insect development, primarily through the dysregulation of juvenile hormone (JH) and 20-hydroxyecdysone (20E), which together orchestrate molting and metamorphosis [12]. JH, a sesquiterpenoid hormone synthesized in the corpus allatum, plays a pivotal role in maintaining larval identity by suppressing precocious metamorphosis in holometabolous insects [13,14,15,16,17]. Consequently, compounds with JH-agonistic or -antagonistic activity have attracted considerable attention as promising insect growth-regulating candidates for insect pest control [18,19].

Several JH analogs, such as methoprene [20], fenoxycarb [21], and pyriproxyfen [22], have been reported as effective IGRs exhibiting JH-agonistic activity. In addition, imidazole-type IGRs—including 1-benzyl-5-[(E)-2,6-dimethyl-1,5-heptadienyl]imidazole (KK-42), which induces precocious metamorphosis in silkworms [23,24], and 1-[3-(4-phenoxyphenoxy)-propyl]imidazole (KS-175) [25], which inhibits ecdysteroid biosynthesis in the prothoracic gland—have demonstrated inhibitory activity in lepidopteran larvae, potentially due to their JH-antagonistic effects [26,27]. The low-molecular-weight, monomeric JH-binding protein (JHBP), identified as a lepidopteran-specific JH transporter, plays a critical role in JH signaling [28,29,30,31,32]. Due to the highly hydrophobic nature of JH, free transport in the hemolymph is inefficient, necessitating carrier proteins such as JHBP to deliver the hormone to target tissues [33]. Given its essential role in JH transport and signaling, JHBP represents a promising molecular target for the development of novel IGRs.

Recently, benzimidazole derivatives have attracted significant attention in both agrochemical and pharmaceutical research due to their diverse and valuable pharmacophoric properties [34]. Also, our research group previously reported that 2-methylbenzimidazole derivatives (MBIs) exhibit insect growth-regulating activity against the silkworm, *Bombyx mori* [27]. Among them, 1-benzyl-2-methylbenzimidazole (BMBI) derivatives have demonstrated dual biological activities—namely, endocrine disruption leading to developmental inhibition and acute toxicity—depending on the substitution pattern on the 1-benzyl moiety [27]. Building on these findings, we synthesized the potential three series of BMBIs bearing alkyl, alkoxy, and alkylamino substituents at the 4′-position of the 1-benzyl group and evaluated their in vivo biological activities.

To elucidate the molecular mechanism of BMBIs, we selected JHBP as a potential target and assessed the interactions between BMBIs and JHBP using a Förster resonance energy transfer (FRET)-based assay. This technique enables direct, quantitative analysis of biomolecular interactions. Specifically, we employed the FRET JH Indicator Agent (FREJIA), a genetically encoded biosensor based on *Bombyx mori* JHBP, which undergoes ligand-induced conformational changes that modulate energy transfer between donor and acceptor fluorescent proteins fused to its termini [35]. Increase in the FRET emission ratio was observed ligand-dependently, with a dissociation constant of 293 nM for JH III and high specificity toward JH-related compounds [35].

Furthermore, X-ray crystallographic analyses of JHBP in both its apo form and in complex with JH II have provided mechanistic insights into ligand recognition and revealed significant conformational changes within the active site upon ligand binding. The comparison of the apo- and JH-bound structures suggests that the uptake and release of JH are controlled by the opening and closing of an α-helix within the JH-binding pocket. This α-helix functions as a gate that facilitates the detection of ligand entry. The apo form of JHBP in its crystalline state features a gate that remains in an open conformation, facilitating access to JH at the retained hormone-binding site. The position of the α-helix in the apo-JHBP structure, resulting in a broad and open conformation, is fully exposed to the solvent area. Further, the orientation of α-helix changes significantly due to JH binding, rotating about 70 degrees toward the pocket compared to the position of the corresponding helix in the apo-structure [36]. To further investigate the binding modes of the newly synthesized BMBI derivatives, we performed X-ray crystallography and molecular docking simulations. These computational approaches, widely used in modern drug discovery, enabled the prediction of binding affinities and the evaluation of complex stability under physiological conditions, thereby supporting the rational design of novel insecticidal agents [37,38].

## 2. Materials and Methods

### 2.1. Materials and Physical Measurements

All reagents were acquired and utilized as received, without undergoing further purification. The components of the mixture were monitored using thin layer chromatography (TLC silica gel 60 F254 Aluminum sheets 20 × 20 cm) and visualized using a combination of UV illumination and spraying with potassium permanganate solution. The purification of the synthesized compounds was performed by gravity column chromatography on silica gel (Wakogel^®^ C-200, Fujifilm Wako Pure Chemical Corporation, Osaka, Japan). The 1H NMR spectra of the isolated compounds were recorded on a JEOL JNM A-400 FT NMR spectrophotometer (400 MHz, JEOL, Tokyo, Japan) with tetramethyl silane as an internal standard. All mass spectrometric data were acquired using the Orbitrap Velos Pro Hybrid Mass Spectrometer (Ultimate Confidence; Thermo Fisher Scientific, MA, USA). Melting points were determined on the uncalibrated MP-500D apparatus (Yanaco, Kyoto, Japan). The FRET-based activity assay was conducted according to the previously described protocol using the JH sensor (FREJIA), developed by Kikuta et al. [35]. Measurements were performed in a 96-well microplate format with a fluorescence plate reader (SH-9000, Corona Denki Co., Ltd., Ibaraki, Japan).

### 2.2. Synthesis of the BMBI Derivatives

**Scheme 1:**
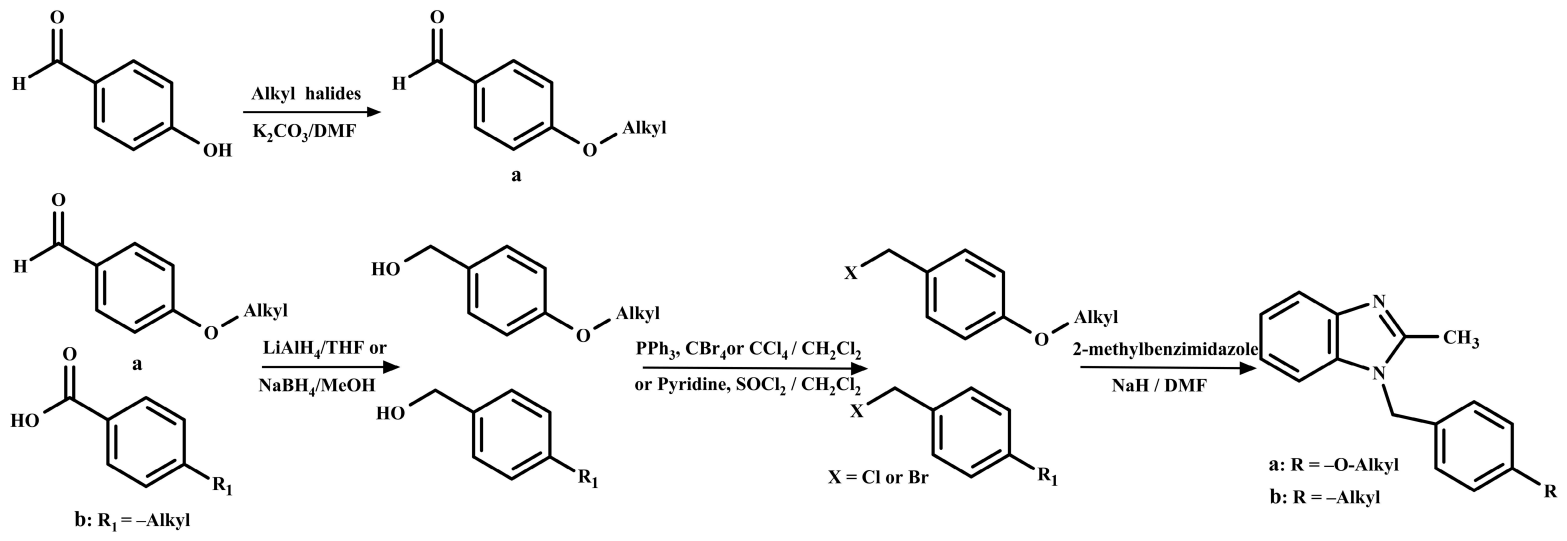
General synthetic route for the preparation of BMBI derivatives.

**Figure 1:**
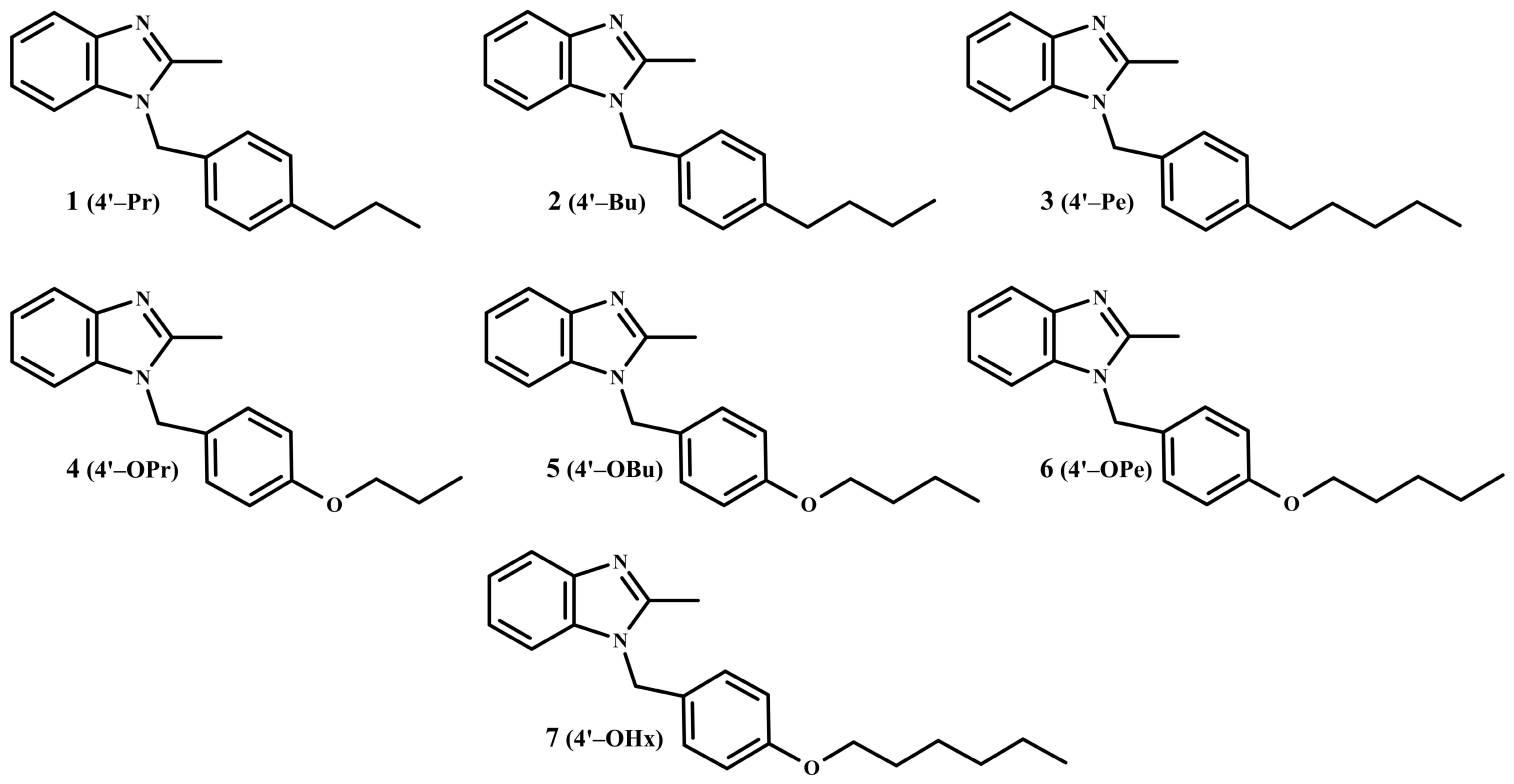
Structures of representative BMBIs used in this study.

All the syntheses were carried out in accordance with the protocol outlined for BMBIs (Scheme 1 and Scheme S1, Figure 1). This synthetic route comprised the reduction of the corresponding heterocyclic carboxylic acid or aldehyde, subsequent transformation of the resulting alcohol into the corresponding halide, and final coupling with 2-methylbenzimidazole via an S_N_2 nucleophilic substitution reaction.

#### 2-Methyl-1-(4-propyloxybenzyl)-benzimidazole (**4** (4′-OPr))

4-Hydroxybenzaldehyde (Tokyo chemical industry Co., Ltd., Tokyo, Japan) (0.53 g, 4.37 mmol) was dissolved in DMF (FUJIFILM Wako pure chemical corporation, Osaka, Japan) (5 mL), followed by the sequential addition of K_2_CO_3_ (FUJIFILM Wako pure chemical corporation) (0.81 g, 5.84 mmol) and 1-bromopropane (FUJIFILM Wako pure chemical corporation) (0.75 g, 6.10 mmol). The mixture was then stirred to give 4-propyloxybenzaldehyde (0.63 g, 88%). The resulting 4-propyloxybenzaldehyde (0.93 g, 5.64 mmol) was dissolved in MeOH (FUJIFILM Wako pure chemical corporation) (5 mL) and reduced to the corresponding benzyl alcohol by treatment with NaBH_4_ (Tokyo Chemical Industry Co., Ltd., Tokyo, Japan) (0.23 g, 6.16 mmol) under overnight stirring, affording 4-propyloxybenzyl alcohol (1.05 g, 89%). The 4-propyloxybenzyl alcohol (0.53 g, 3.16 mmol) in DCM (FUJIFILM Wako pure chemical corporation) (15 mL) was treated with pyridine (FUJIFILM Wako pure chemical corporation) (9.48 mmol) and thionyl chloride (FUJIFILM Wako pure chemical corporation) (0.46 g, 3.87 mmol) at 0 °C, converted to 4-propyloxybenzyl chloride (0.43 g, 73%). To obtain the **4** (4′-OPr), 4-propyloxybenyl chloride was subjected to an S_N_2 reaction in which 2-methylbenzimidazole (Tokyo Chemical ndustry Co., Ltd., Tokyo, Japan) (0.40 g, 3.03 mmol) in DMF (4 mL) was deprotonated with NaH (FUJIFILM Wako pure chemical corporation) (0.10 g, 2.08 mmol, 50% oil suspension), followed by the addition of benzyl chloride electrophile (0.43 g, 2.33 mmol) to afford the corresponding coupled product. The reaction mixture was purified by silica gel chromatography to obtain the **4** (4′-OPr) as a colorless crystal (0.53 g, 81%); m.p. 69.9–71.3 °C; ^1^H-NMR δH (400 MHz, CDCl_3_): 7.72–7.70 (m, 1H), 7.25–7.17 (m, 1H), 6.99–6.97 (m, 2H), 6.83–6.79 (m, 2H), 5.26 (s, 2H), 3.87 (t, J = 6.6 Hz, 2H), 2.57 (s, 3H), 1.82–1.71 (m, 2H), 1.01 (t, J = 7.3 Hz, 3H); HRMS m/z [M + H]^+^: calcd for C_18_H_21_ON_2_: 281.16484; Found, 281.1648. Other BMBIs syntheses are detailed in the Supplementary File.

### 2.3. Assessment of Biological Efficacy of BMBIs

The biological activity against *Bombyx mori* (Shunrei × Shougetsu strain, Ueda-Sanshu) (Lepidoptera, Bombycidea) was evaluated using larvae reared on Silkmate 2S (Nippon-nosan, Kogyo, Japan) under controlled environmental conditions, comprising a 12 h light/12 h dark photoperiod at 25 °C. 10 μg of BMBIs in 1 µL, dissolved in acetone, (FUJIFILM Wako pure chemical corporation) were topically applied to the dorsal thorax of one-day-old 3rd instar larvae, while control groups received acetone alone. Five to ten larvae were used in triplicates for the assay, and their activity was assessed based on two different symptoms: “acute toxicity,” which refers to death within 48 h after application, and “growth inhibition,” which refers to death occurring after 48 h. Larval mortality was observed over a period of 30 days, and the effects of acute toxicity, molting inhibition, precocious metamorphosis and pupal inhibition were recorded separately. The Abbott’s formula (Abbott, 1925) [39] was used to correct mortality rates, which were assessed on a percentage scale ranging from 0 to 100.

Corrected mortality (%) = (P − Po)/(100 − Po) ×100

P represents the proportion of mortality observed in treated insects, whereas P_0_ denotes the percentage mortality recorded in the untreated control group.

### 2.4. Fluorometric FRET Bioassay

FREJIA, a recombinant *Bombyx mori* JHBP conjugated to two distinct fluorescent chromophores (mTFP1 and mVenus), was employed as a JH sensor to evaluate the interaction of the test compounds with JHBP through a FRET-based activity assay [35]. To measure fluorescence readings, each well of a 96-well microplate (Nunc™ MicroWell™ 96-Well, Nunclon Delta-Treated, Flat-Bottom Microplate, Thermo Fisher Scientific) was sequentially loaded with FREJIA (at a final concentration of 1 µM, 297 μL) in 10 mM Tris–HCl buffer (containing 150 mM NaCl (pH 7.5), followed by the addition of BMBIs or JH III (10–1000 µM, 3 μL) dissolved in ethanol. Fluorescence emission at 530 nm was measured at the corresponding excitation wavelength using a fluorescence plate reader (SH-9000, Corona Denki Co., Ltd., Ibaraki, Japan). 10 μM JH III prepared in ethanol served as the negative control, whereas ethanol was utilized as the positive control. FRET activity values were calculated from the fluorescence intensity ratio (% of 494 nm/530 nm) using the following equation.

FRET activity rate (%) = (Ft − Fc)/(Fj − Fc) ×100

In this equation, Ft represents the FRET activity following the addition of the test compound, Fc denotes the negative control, and Fj is the positive control.

The concentration-response curve was generated using OriginPro 2026 (64-bit) SR1 10.3.0.197 (Learning Edition) data analysis software.

### 2.5. X-Ray Crystallography

Ligand-bound JHBP complexes were prepared as described previously [36]. Briefly, GST-fused recombinant JHBP was expressed in *Escherichia coli* BL21(DE3) cells and purified to homogeneity using a GSTrap column followed by a HiLoad 26/60 Superdex 75 pg column. The purified protein was incubated with an excess of **2** (4′-Bu) or **4** (4′-OPr) to form the corresponding complexes. The complexes were subsequently concentrated to 10 mg mL^−1^ and used for crystallization.

Crystallization screening of the ligand–JHBP complexes was performed at 20 °C using the sitting-drop vapor-diffusion method. Crystals of the **2** (4′-Bu)-bound JHBP complex were obtained from a reservoir solution containing 0.1 M sodium acetate (FUJIFILM Wako Pure Chemical Corporation) (pH 5.6), 1.7 M ammonium sulfate (Nacalai Tesque, Kyoto, Japan), and 3% (*w/v*) 1,6-hexanediol (FUJIFILM Wako Pure Chemical Corporation). Crystals of the **4** (4′-OPr)-bound JHBP complex were obtained from a reservoir solution containing 0.1 M sodium acetate (pH 5.2) and 1.7 M ammonium sulfate. Diffraction experiments for JHBP in complex with **2** (4′-Bu) and **4** (4′-OPr) were conducted at the beamlines of Photon Factory (PF) or Photon Factory Advanced Ring (PF-AR), High Energy Accelerator Research Organization, Tsukuba, Japan (Table S1). The data were collected using a PILATUS 2M detector (Dectris Ltd., Baden-Daettwil, Switzerland) and integrated and scaled using HKL2000 v720 [40] and XDS (BUILT=20190315) [41].

The crystal structure was determined using the molecular replacement method with MOLREP v7.0 [42] in the CCP4 program suite v9.0 [43], using the structure of JHBP complexed with JH-II (PDB code 3AOS) [36] as the starting model. Manual model building and molecular refinement were performed using Coot v0.9.8 [44,45] and REFMAC5 v5.8 [46]. Stereochemistry of the models was analyzed using Rampage (http://www-cryst.bioc.cam.ac.uk/rampage, accessed on 17 June 2026) [47].

### 2.6. Validation of Docking Simulation Method

As with any computational methodology, docking protocols require systematic validation to ensure the reliability and accuracy of the predicted binding modes [48]. The docking protocol employed in this study was validated using a redocking approach, in which pose selection was performed at the receptor’s active site where the native ligand binds. Mock docking was performed using the X-ray crystallographic structure of the silkworm *Bombyx mori* JHBP (PDB: 3AOS), which was co-crystallized with the control ligand, JH II. The co-crystallized ligand and water molecules were removed prior to docking, and the active site of the target protein was subsequently predicted using PyMOL (version 3.1.6.1). AutoDock Tools 1.5.7 was employed to generate PDBQT files for both JHBP and ligand. The protein was subsequently prepared by the addition of polar hydrogen atoms and the assignment of Kollman united-atom charges, while full rotational flexibility about ligand single bonds was permitted during docking. Docking simulations were performed using AutoDock Vina 1.1.2 (Scripps Research institute, San Diego, CA, USA). This procedure was repeated multiple times to ensure the robustness and reproducibility of the docking results. The ligand–protein interactions were visualized in both two- and three-dimensional representations using the BIOVIA Discovery Studio 2025 and PyMOL 3.1.6.1. The docked binding conformation obtained from each scoring function was compared with the experimentally determined conformation observed in the protein–ligand complex derived from the X-ray crystallographic structure. An RMSD value of ≤2.0 Å relative to the native binding conformation of the control ligand is generally considered an acceptable criterion and indicates the reliability of the docking protocol. The optimal binding conformation was selected for further analysis based on the molecular docking score.

### 2.7. Molecular Docking Simulation

To further elucidate the structure–activity relationships (SAR) and the potential mechanism of action of the synthesized compounds, the binding modes of two randomly selected, highly active derivatives, **2** (4′-Bu) and **4** (4′-OPr), with JHBP (PDB: 3AOS) were investigated using AutoDock Vina. The three-dimensional atomic coordinates of the selected compounds were constructed using MarvinSketch 25.3.6, and the resulting structures were subsequently employed for molecular docking simulations. The 3D crystallographic structure of the target JHBP (PDB ID: 3AOS) was retrieved from the Protein Data Bank, and the JH II and water were subsequently removed prior to analysis. The **2** (4′-Bu) and **4** (4′-OPr) were docked into the predicted active site of the target JHBP using the same grid box coordinates for all complexes and the methods described above to analyze their binding interactions in silico and to compare their activity.

## 3. Results

### 3.1. Biological Activity of BMBIs

**Figure 2:**
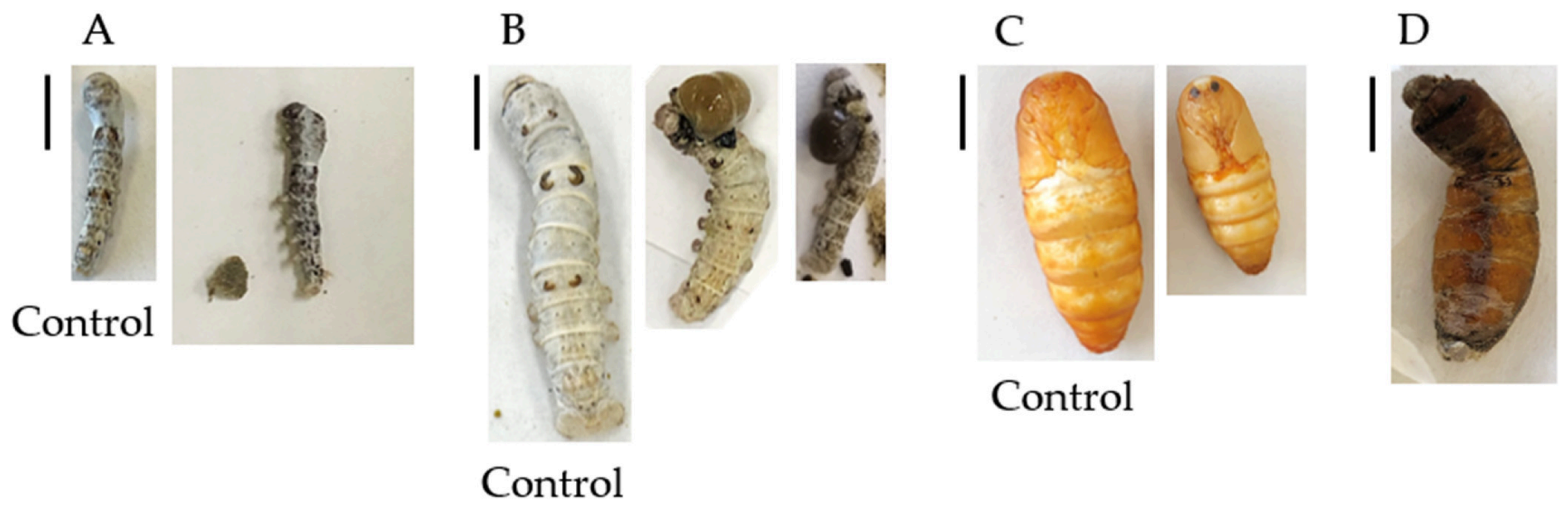
Phenotypic characteristics observed after treatment of 3rd instar with BMBIs. Day one 3rd instar *B. mori* larvae were topically treated with BMBIs dissolved in acetone and their phenotypes were observed. The bars in the upper-left corner of each picture represent 1 cm; (**A**) Representative control and larva showing acute toxicity induced by **2** (4′-Bu, 10 µg/larva); (**B**) Representative control and larva exhibiting cuticular abnormality induced by **4** (4′-OPr, 10 µg/larva); (**C**) Representative control and pupa showing precocious metamorphosis induced by **4** (4′-OPr, 10 µg/larva); (**D**) Representative larva exhibiting pupation impairment induced by **4** (4′-OPr, 10 µg/larva, right).

Treatment of third-instar *B. mori* larvae on the first day with synthesized BMBIs resulted in diverse biological effects, including acute toxicity and growth inhibition (Figure 2). Larvae showing acute toxicity died within 48 h of treatment without notable morphological changes, (Figure 2A) whereas those exhibiting growth inhibition died at later time points and frequently displayed disruptions in developmental processes such as molting or pupation (Figure 2B–D).

The biological effects of BMBIs varied depending on the nature of their substituents. BMBIs bearing 4′-alkyl or alkoxy group tended to induce acute toxicity or growth inhibition, respectively, whereas those containing a 4′-alkylamino group exhibited comparatively low biological activity.

**Figure 3:**
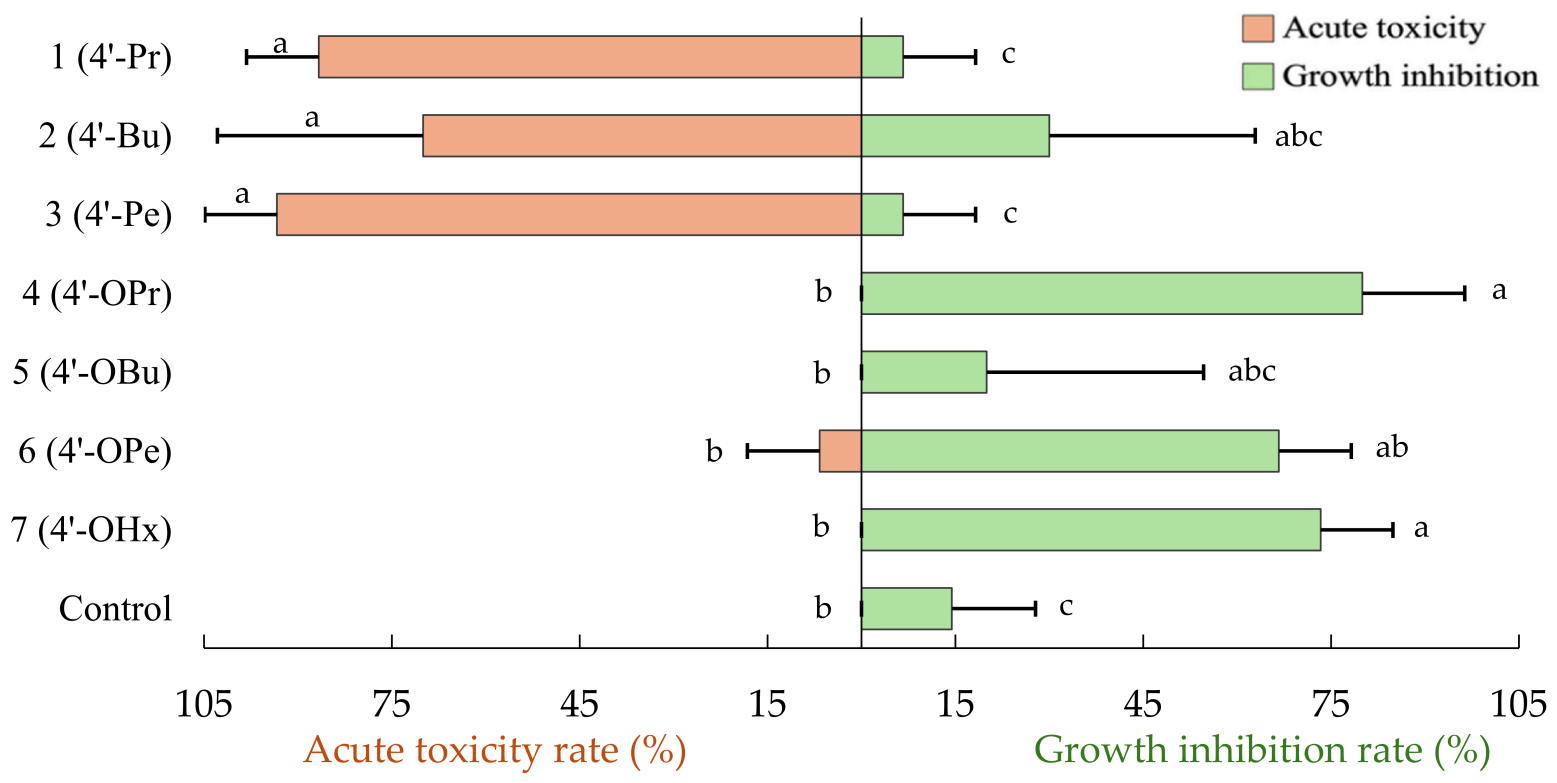
Biological activity of 4′-alkyl and 4′-alkoxy BMBIs on day one 3rd instar larvae at 10 µg/larva. The horizontal error bars denote S.D. Day one 3rd instar *B. mori* larvae were topically treated with BMBIs dissolved in acetone. Acetone was used as a control. Each assay was performed with a total of 15–55 larvae. Orange bars indicate acute mortality rate; Light green bars indicate growth inhibition rate. The x and y axes represent mortality rate (%) and compound type, respectively. Statistical significance was evaluated using one-way ANOVA followed by Games–Howell multiple comparisons test. Differences were considered statistically significant at *p* < 0.05. Different lowercase letters (a, b and c) indicate statistically significant differences among compounds (compounds sharing the same letter are not significantly different) based on the multiple comparison test, performed separately for acute toxicity rate and growth inhibition rate. The x-axis was extended to 105% to fully display error bars, although the maximum possible mortality is 100%.

**Figure 4:**
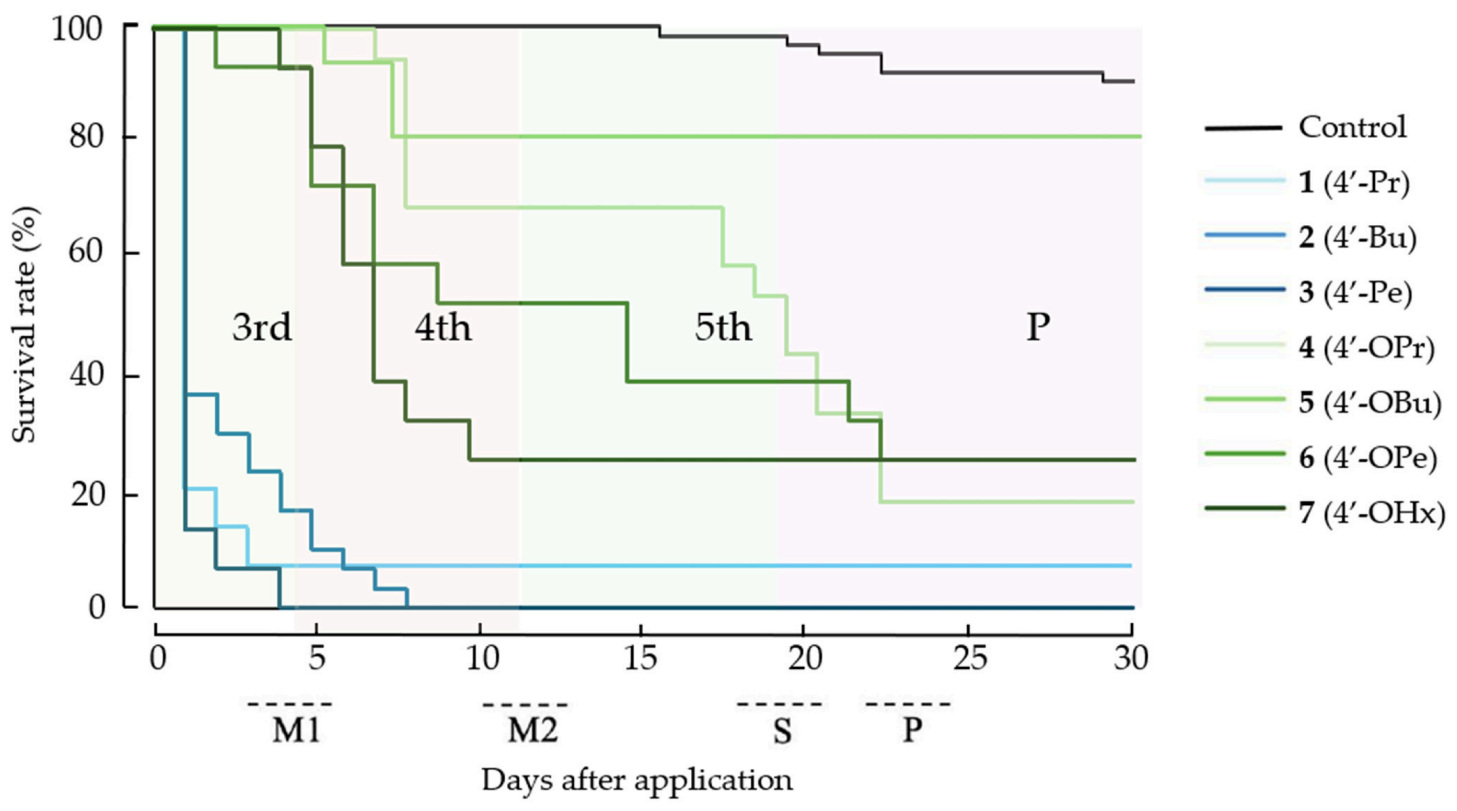
Survival curves after administration of 10 µg/larva of 4′-alkyl and 4′-alkoxy BMBIs on day one 3rd instar larvae. Day one 3rd instar *B. mori* larvae were topically treated with BMBIs dissolved in acetone, and survival rates were observed for 30 days after treatment. Acetone was used as a control. Each assay was performed with a total of 15–55 larvae. Data represents means of survival rates. The x and y axes represent days after application and survival rate (%), respectively. The letters below the figure indicate the developmental stages of control larvae; M1 = molting from 3rd to 4th instar, M2 = molting from 4th to 5th, S = spinning, P = pupation. Colored areas in the figure denote durations of each instar; yellow = duration of 3rd instar, orange = duration of 4th instar, green = duration of 5th instar, and red = pupal period.

Alkyl-substituted BMBIs exhibited pronounced larvicidal activity, achieving ≥70.0% mortality (Table 1, Figure 3), leading rapid lethality after the compound administration (Figure 4). Alkoxy-substituted BMBIs caused developmental disruption rather than acute toxicity (Table 1, Figure 3), predominantly reducing survival rates during molting from 3rd to 4th instar, from 4th to 5th, and during pupation (Figure 4). In contrast, alkylamino-substituted BMBIs exhibited relatively weak larvicidal activity, with ≤33.33% mortality (Table S2, Figure S2), primarily through interference of pupation. In addition, previous studies conducted by our research group demonstrated that methoprene and KK-42 exhibited moderate biological activity, inducing approximately 50% mortality by growth inhibition and acute toxicity against third-instar larvae respectively [27]. Thus, these findings demonstrate that the newly synthesized derivatives possess substantially enhanced biological activity relative to previously reported analogues. Among the tested compounds, **4** (4′-OPr) elicited the highest growth-inhibitory rate (80.0%) (Table 1, Figure 3), accompanied by multiple developmental impairments, including molting disruption, cuticular abnormality (Figure 2B), precocious metamorphosis (Figure 2C), and pupation inhibition (Figure 2D). Although both **2** (4′-Bu) and **9** (4′-NPr) possess benzyl substituents of the same length as **4** (4′-OPr), their biological activities differed markedly. The **2** (4′-Bu) exhibited 70.0% acute lethality and 30.0% growth inhibition, whereas **9** (4′-NPr) showed only 13.33% growth inhibition (Table S2, Figure S2). Furthermore, these compounds induced lethality at different time points consistent with their biological effects: **4** (4′-OPr) induced lethality primarily during molting or pupation, **2** (4′-Bu) within 48 h after treatment, and **9** (4′-NPr) only a slight decrease survival during pupation (Figure S3).

### 3.2. Binding Affinity of BMBIs with JHBP

**Figure 5:**
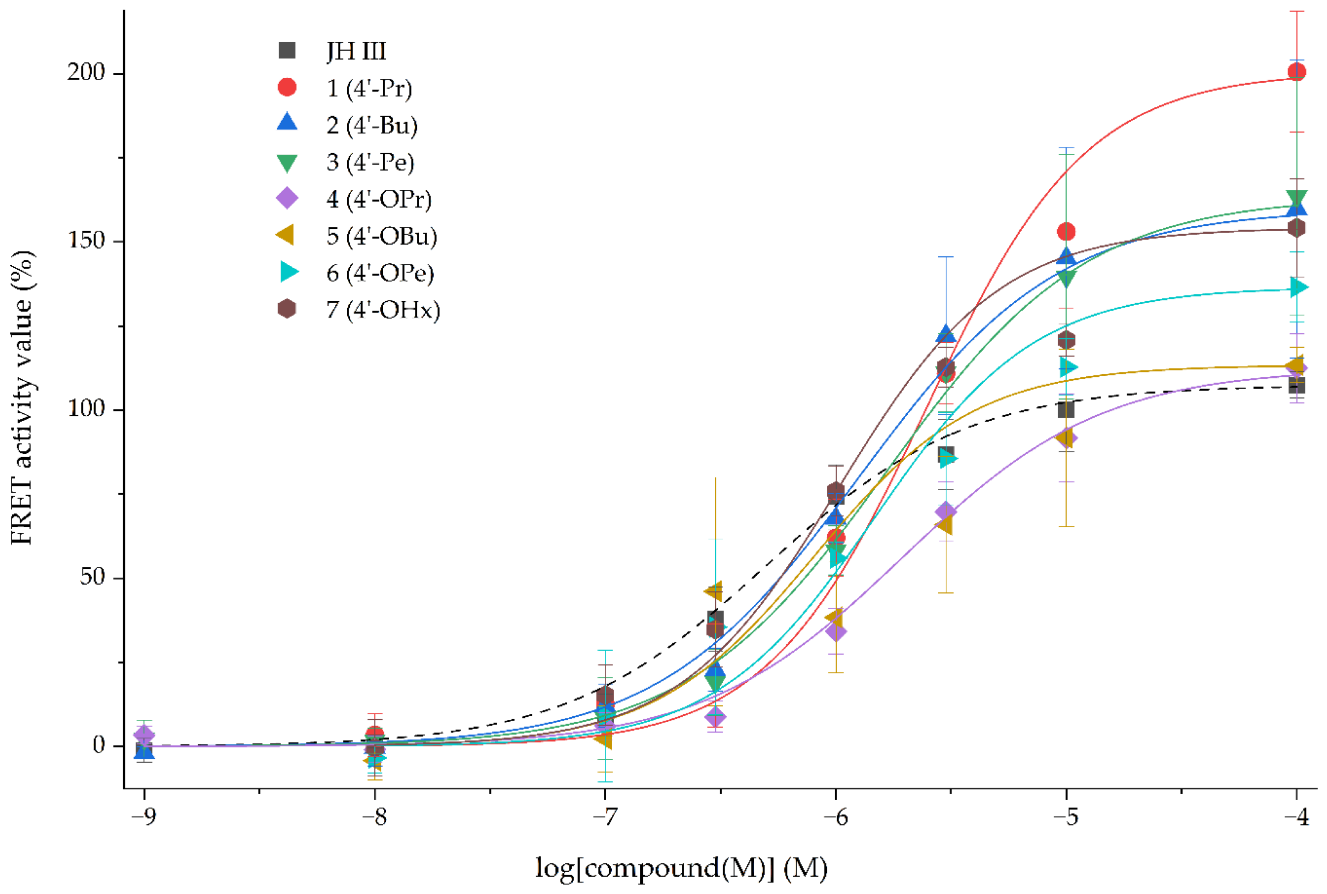
Dose-response curve of 4′-alkyl and 4′-alkoxy BMBIs on FRET activity. Data represents mean ± S.D. Each assay was performed at least in triplicate.

To investigate the relationship between the biological activity of the synthesized BMBIs and JHBP, their binding affinities to JHBP were evaluated using the FREJIA method [35]. Half-maximal effective concentration (EC_50_) values were determined from the dose–response relationships derived from the FRET results, which exhibited characteristic sigmoidal profiles, indicating a concentration-dependent increase in biological activity that approached a plateau at higher concentrations (Figure 5 and Figure S4). Based on the FRET assay, JH III, the natural ligand of JHBP, demonstrated an EC_50_ value of 0.55 ± 0.08 µM. Among the tested compounds, all BMBIs bearing 4′-alkyl or 4′-alkoxy groups exhibited consistent binding affinities, which were comparable to JH III (Table 2). Notably, BMBIs with potent biological activity, namely **4** (4′-OPr) and **2** (4′-Bu), showed JHBP binding affinities with EC_50_ values of 2.08 ± 0.42 µM and 1.22 ± 0.15 µM, respectively. The **2** (4′-Bu) showed the strongest JHBP binding among BMBIs possessing 4′-alkyl group, followed by 4′-Pe (**3**, EC_50_ = 1.63 ± 0.22 µM) and 4′-Pr (**1**, EC_50_ = 2.75 ± 0.42 µM). Furthermore, in the 4′-alkoxy containing group, **7** (4′-OHx) exhibited the highest binding affinity with the EC_50_ value of 1.07 ± 0.19 µM, and 4′-OBu (**5**, EC_50_ = 2.10 ± 0.80 µM) and 4′-OPe (**6**, EC_50_ = 1.65 ± 0.39 µM) also exhibited moderate binding affinity.

By contrast, BMBIs bearing 4′-alkylamino groups showed relatively weaker interactions with JHBP. Within this group, **11** (4′-NHx) displayed a comparatively higher affinity (EC_50_ = 2.14 ± 0.21 µM), whereas the others exhibited much lower binding affinities: **8** (4′-NEt), 10.09 ± 1.96 µM; **9** (4′-NPr), 6.79 ± 3.06 µM; and **10** (4′-NPe), 4.64 ± 1.38 µM (Table S3). Overall, 4′-alkyl and 4′-alkoxy BMBIs, which demonstrated potent larvicidal activity, exhibited favorable binding affinities to JHBP, suggesting that interaction with JHBP may contribute to their biological efficacy.

### 3.3. Crystal Structures and Binding Modes of BMBIs in JHBP

**Figure 6:**
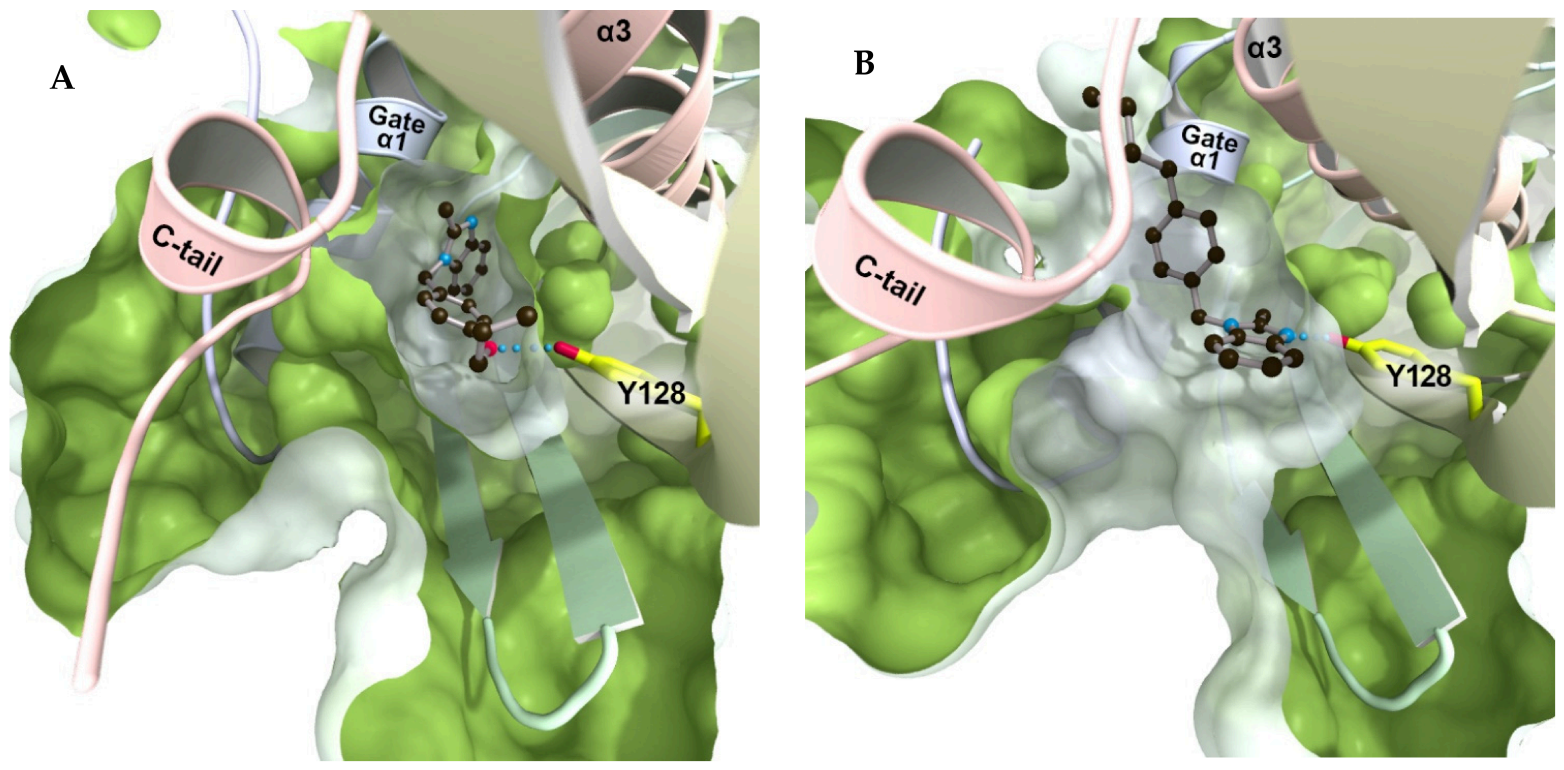
Structural view of the JH-binding pocket in the crystal structures of the Silkworm *Bombyx mori* JHBP–BMBI complexes. The molecular surfaces of BMBI–bound JHBPs are vertically sectioned to provide a perpendicular view of the JH-binding pocket. The internal region of the protein is colored green, whereas the external surface and the interior of the binding pocket are colored grey. The bound BMBI derivatives are represented in ball-and-stick models, with oxygen and nitrogen atoms colored red and blue, respectively. The side chain of Tyr128, a key interacting residue, is depicted as a stick model, and hydrogen bonds are depicted as light-blue dashed lines. (**A**) View of the JH-binding pocket in the crystal structure of the JHBP-**4** complex. (**B**) View of the JH-binding pocket in the crystal structure of the JHBP-**2** complex.

The crystal structures of the **4** (4′-OPr)-bound and **2** (4′-Bu)-bound JHBP complexes were determined at resolutions of 1.85 and 2.40 Å, respectively. In both structures, well-defined electron density corresponding to the bound ligand was observed within the JH-binding pocket, allowing reliable modeling of the ligands (Figure 6). Based on the crystal structures, both derivatives bind within the JH-binding pocket; however, pronounced conformational changes are observed. In the JHBP–**4** complex (Figure 6A), the co-crystallized ligand is positioned within the interior of the binding pocket buried inside the JHBP molecule. In contrast, in the JHBP–**2** complex (Figure 6B), the pocket exhibits an outward open conformation on both sides. This structural rearrangement is induced by positional shifts of the N-terminal arm (N-terminus) and the C-terminal tail (comprising the C-terminus and the 3_10_ helix), which are connected via hydrogen-bonding interactions, together with the gate helix α1. As a result, the hydrophobic alkylbenzyl moiety and the benzene ring on the benzimidazole side of **2** (4′-Bu) are accommodated near both entrances of the binding pocket. Within this structural context, the hydroxyl group of Tyr128 forms a hydrogen bond with the oxygen atom in the alkoxy group of **4** (4′-OPr) and with the nitrogen atom in the benzimidazole moiety of **2** (4′-Bu), thereby highlighting that the two ligands adopt opposite orientations within the pocket. However, this hydrogen bond accounts for the high enantioselectivity of JHBP.

**Figure 7:**
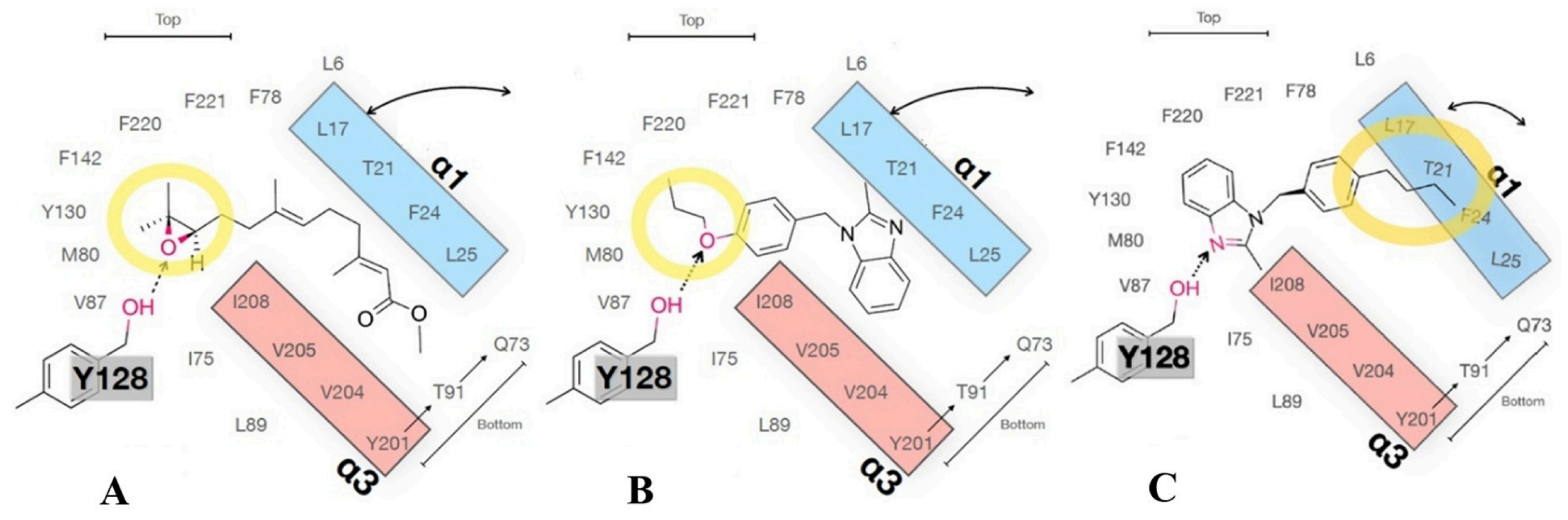
Schematic representation of the binding sites of three ligands within the JHBP, illustrating their binding affinity mechanisms. Polar hydrogen-bond interactions are denoted by dashed arrows, while additional polar and nonpolar interactions are depicted surrounding each ligand. Arrows show the flexible movement of α1 helix. Yellow circles indicate epoxide of JH and substituted group of BMIs. (**A**) Binding interactions of JH III with JHBP [36]. (**B**) Binding interactions of ligand **4** (4′-OPr) with JHBP. (**C**) Binding interactions of ligand **2** (4′-Bu) with JHBP.

The schematic representation (Figure 7) of the crystallographic structures further elucidates the binding-site behavior of the ligands within the complexes. In a previous report, Rintaro Suzuki et al. schematically illustrated the interaction between JH III and JHBP. (Figure 7A). The structure of the JHBP–JH III complex revealed that JHBP recognizes the distinctive conformation and functional groups of JH with high specificity through a combination of hydrophobic interactions and an intricate hydrogen-bonding network. To achieve optimal accommodation within the binding pocket, the JH III molecule adopts an L-shaped conformation, accomplished by a series of successive gauche conformations. Further, previous study identified the significant role of the hydrogen bond interaction between the OH group of Tyr128 and the epoxide oxygen of JH by constructing 23 JHBP single mutants for 20 key interacting residues and examining their JH-binding activity in vitro [36]. In contrast, the structure of the JHBP–**4** complex (Figure 7B) adopts a similar conformation, featuring a hydrogen-bonding interaction between the OH group in Tyr128 residue and the ether oxygen in **4**, and is nearly identical to the conformations observed for JH III. The JHBP–**2** complex (Figure 7C) exhibits a similar interaction with the Tyr128 residue; however, it displays significant conformational differences compared to JH III and **4** (4′-OPr), probably due to the lack of an oxygen atom in the side chain. The complex of JH III, **4** (4′-OPr) and **2** (4′-Bu) share a comparable chemical environment and engage a similar set of interacting residues; however, the JHBP–**2** complex exhibits pronounced differences in the relative orientation of the N-arm, the C-tail and the gate helix α1.

### 3.4. Validation of the Docking Simulation

**Figure 8:**
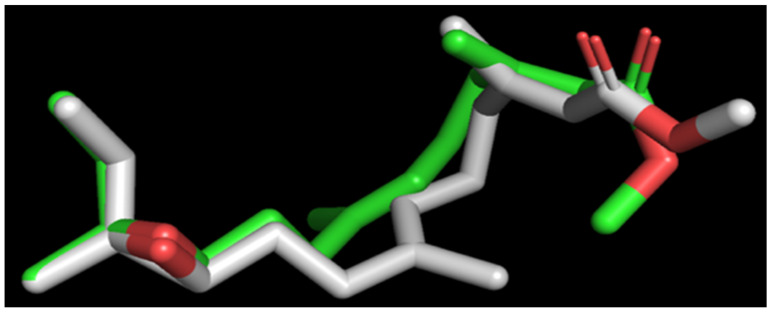
Docking software validation results: the docked configuration (green) was superimposed onto the experimentally determined binding pose obtained by X-ray crystallography (grey). Oxygen atoms are indicated by red color RMSD = 1.420 Å.

The docking protocol was validated by re-docking the co-crystallized JH II into the active binding site of the target JHBP, thereby confirming the reliability and predictive accuracy of the docking methodology. The re-docked pose, with an RMSD of 1.420 Å, reproduced binding within the same active site as the reference ligand with minor deviations in the flexible carbon chain. Tyr128 was identified as a key interacting residue, forming conserved hydrogen-bonding interactions with the ligand in both the docked and the corresponding crystallographic structure. The native co-crystallized ligand was superimposed onto the docked complex, as shown in Figure 8. The reference conformation JH II obtained X-ray crystallographic structure is shown in grey, while the re-docked complex of the JH II within the JHBP active binding site is depicted in green. A low RMSD suggested the validation of the docking procedure.

### 3.5. Molecular Docking Simulation

**Figure 9:**
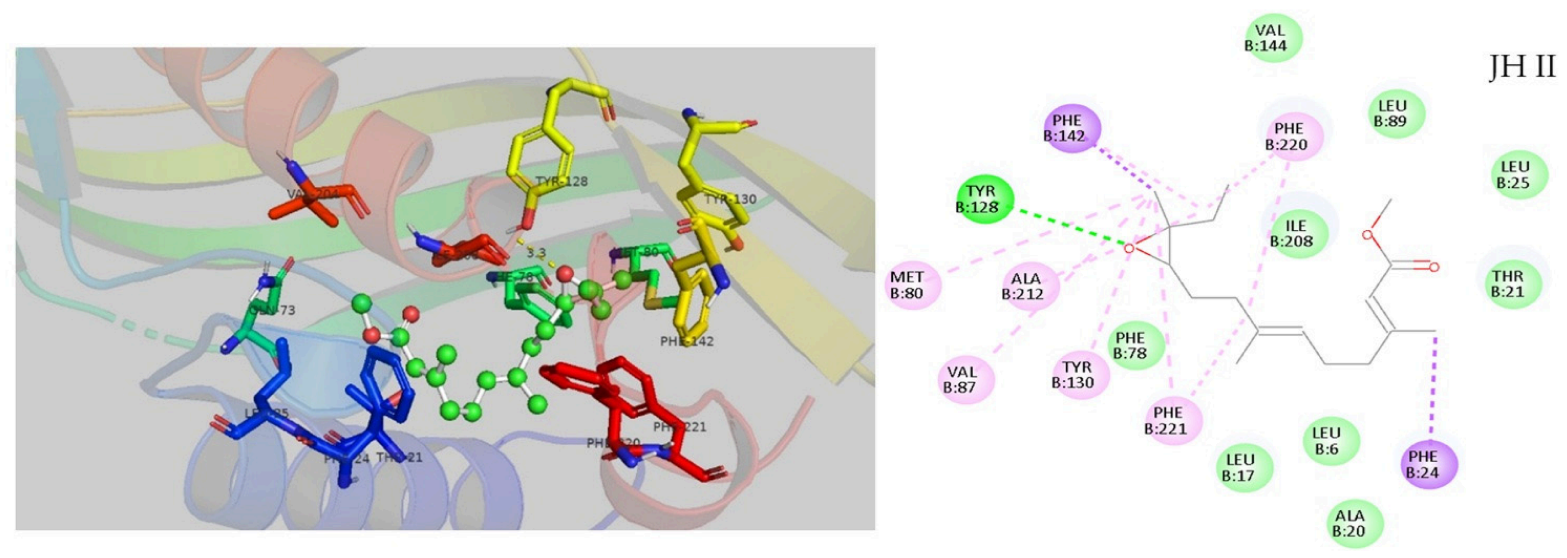

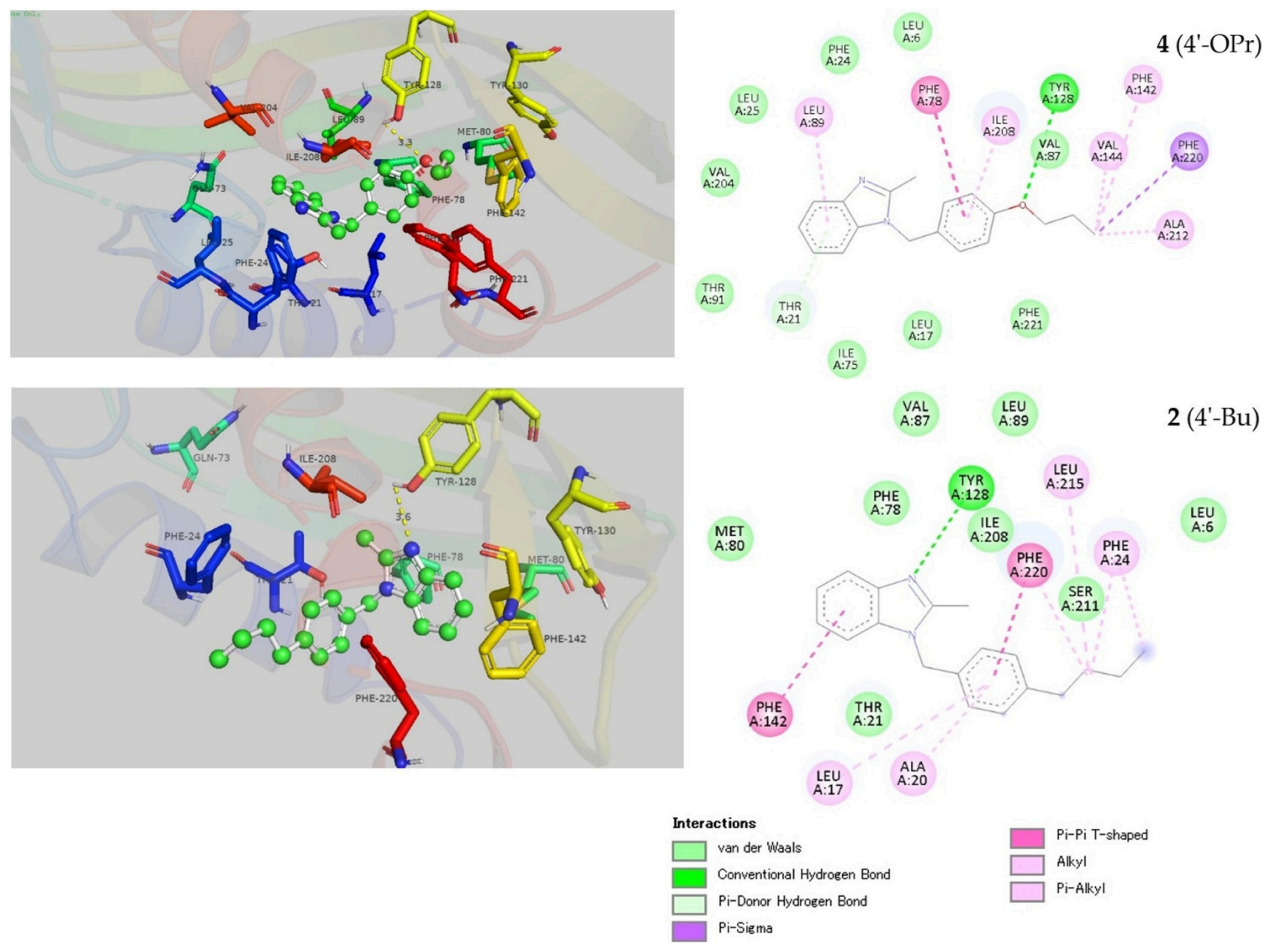
3D (**left**) and 2D (**right**) views of the molecular interactions of amino acid residues of JHBP with JH II, **4** (4′-OPr) and **2** (4′-Bu). In 3D diagram, hydrogen bond interaction is denoted by yellow dash line. Each ligand is represented in a ball-and-stick format, oxygen and nitrogen atoms are depicted in red and blue, respectively. In 2D diagram, the amino acid residues participating in ligand binding within the active site are highlighted by circles. Residues involved in van der Waals interactions are denoted by light green circles, hydrogen bond interaction is green, those participating in pi–alkyl/alkyl interactions by pink circles, those engaged in pi–pi T-shaped interactions by magenta circles and pi–sigma by violet circles.

To gain mechanistic insights into the mode of action for the observed phenotypic differences, we conducted a comparative analysis of **4** (4′-OPr) and **2** (4′-Bu) along with native ligand (JH II) by docking simulations. The docking simulations were conducted using the JHBP crystal structure of silkworm *Bombyx mori* (PDB ID: 3AOS), employing the AutoDock Vina software. Among the compounds tested, **4** (4′-OPr) showed highest growth development inhibition, and **2** (4′-Bu) demonstrated highest acute toxicity against *B. mori.* Therefore, we performed in silico re-docking studies based on the available co-crystal structures of JH II, **4** (4′-OPr) and **2** (4′-Bu) to predict potential interaction modes, generated multiple docking poses, and selected the most energetically favorable conformation for further analysis (Figure S5). Three-dimensional docking poses depicting the binding modes of the compounds with JHBP, together with corresponding two-dimensional diagrams illustrating residue–ligand interactions, are presented in Figure 9; the estimated binding affinities (kcal/mol) and key interacting residues are summarized in Table 3. 

**Figure 10:**
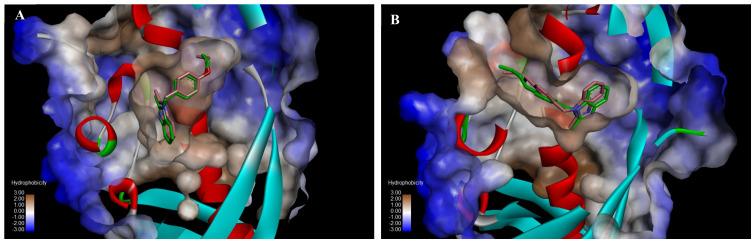
The crystallographic conformation of crystal structure of new derivatives and the docked conformations was compared through structural superposition of the protein coordinates. The molecular surface of the JHBP active site was color-coded according to the hydrophobic surface of the constituent amino acid residues. (**A**) The surface structure of active binding pocket of JHBP-**4** complex, co-crystal structure is denoted by green color and re-docking structure by pink color. (**B**) The surface structure of active binding pocket of JHBP-**2** complex, co-crystal structure is denoted by green color and re-docking structure by pink color.

The native ligand, JH II, exhibited a docking pose within the active site that closely recapitulates the crystal structure, revealing a diverse array of interactions within the JHBP binding pocket. The H-bond interactions play important roles in the interaction of epoxy group of JH II with the Tyr128 residues in the active site of JHBP, thereby contributing to both binding selectivity and stabilization of the L-shaped conformation. Furthermore, this complex is stabilized by pi-sigma interactions involving the methyl group on the epoxide side with Phe142, as well as the methyl group on the ester side with Phe24 amino acid residues. In addition to these interactions, JH II also engages in alkyl and pi–alkyl interactions with residues such as Met80, Val87, Tyr130, Ala212, Phe220, Phe221, and Phe142, along with extensive van der Waals interactions. The binding models indicate that compounds **4** (4′-OPr) and **2** (4′-Bu) engage the same active site of JHBP as the native JH II ligand, mediated by a combination of hydrogen-bonding, hydrophobic, electrostatic, and van der Waals interactions. Notably, both the JHBP–**4** and JHBP–**2** complexes exhibit a hydrogen-bonding interaction with the Tyr128 residue, similar to that observed in the JHBP–JH complex; however, opposite conformations are evident between **2** (4′-Bu) and **4** (4′-OPr), a finding that is consistent with crystallographic observations. In addition, the **4** (4′-OPr) complex is further stabilized by a pi-donor hydrogen bond between the benzimidazole phenyl ring and Thr21, as well as by a pi-pi T-shaped interaction between the phenyl ring of the alkoxy group and the Phe78 residue. The **2** (4′-Bu) complex exhibits pi–pi T-shaped interactions between the benzimidazole phenyl ring and Phe142, as well as between the phenyl ring of the alkylbenzyl moiety and the Phe220 residue, while the hydrophobic alkylbenzyl moiety is positioned to engage in additional hydrophobic interactions. In contrast, both JH II and **4** (4′-OPr) exhibit comparable hydrogen-bond distances (d = 3.3 Å), whereas **2** (4′-Bu) shows a slightly longer distance (d = 3.6 Å); correspondingly, the binding affinities of JH II, **4** (4′-OPr), and **2** (4′-Bu) are −8.4, −9.1, and −8.2 kcal/mol, respectively. Furthermore, comparison of the optimal docking models of **4** (4′-OPr) and **2** (4′-Bu) with the corresponding crystal structures confirms that the ligands adopt the correct positioning and orientation within the binding pocket, as validated by structural superimposition of the protein coordinates (Figure 10).

## 4. Discussion

MBI derivatives represent a promising class of IGRs, as previously reported by our group [23,25,27,49,50]. Building on those studies, we synthesized a new series of BMBI derivatives bearing alkyl, alkoxy, or alkylamino substituents at the 4′-position of the benzyl ring to probe how electron donating character and steric variation influence biological activity (Figure 1). We evaluated these compounds against third instar *Bombyx mori* larvae and quantified ligand binding using a FRET based JHBP sensor. Compounds that produced distinctive phenotypes—most notably **4** (4′-OPr) and **2** (4′-Bu)—were advanced to X ray crystallography and molecular modeling.

Overall, most substituents conferred measurable activity that clustered into two principal outcomes: (i) acute lethality (death within 48 h) and (ii) developmental disruption, including molting failure and pupation inhibition. Alkyl substituted derivatives predominantly induced acute toxicity with only modest growth inhibition, whereas alkoxy substituted derivatives mainly produced developmental defects and pupation failure. Alkylamino substituted derivatives were generally weakly active, showing limited acute toxicity or marginal growth inhibition. Within the alkoxy series, the dominant phenotype was growth inhibition associated with molting failure; pupation inhibition occurred less frequently. This phenotypic diversity suggests that BMBIs may act through multiple molecular targets or mechanistic pathways rather than a single unified mode of action. Previous studies have shown that imidazole-type IGRs such as KK-42 [19,26] and KS-175 [25], inhibit lepidopteran larval development, an effect that has been attributed in part to JH antagonism [26,27] and is associated with phenotypes such as precocious or incomplete metamorphosis, morphological abnormalities, and cuticular defects [51,52]. To clarify the mechanisms underlying the phenotypes observed here, assessment of JH agonistic or antagonistic activity is therefore essential [19].

To explore whether these effects involve perturbation of JH transport by low-molecular-weight JH binding protein (JHBP, ≈30 kDa) [19,36], we measured ligand affinity using a FRET based JHBP sensor [35]. In this assay, JH III displayed an EC_50_ of 0.55 ± 0.08 µM. Alkoxy and alkyl substituted derivatives generally showed enhanced binding affinity with EC_50_ values comparable to JH III, whereas alkylamino derivatives exhibited substantially weaker binding. Binding affinity correlated well with biological activity for the alkoxy and alkylamino series; however, several alkyls substituted compounds produced strong biological effects despite only modest differences in measured affinity, indicating that additional targets effects may contribute to their acute toxicity. Across the three substituent classes, we observed clear class dependent trends but no consistent relationship between side chain length and either binding affinity or phenotype.

To elucidate the structural bases for the divergent phenotypes of alkyl versus alkoxy substituents, we solved crystal structures and performed docking for **4** (4′-OPr) and **2** (4′-Bu). Prior structural studies identified the α1 helix at the entrance of the JHBP pocket as a dynamic gating element that adopts distinct orientations in the gate open apo form and the gate closed ligand bound form [36]. Moreover, JHBP from *Galleria mellonella* has been reported to assume a gate-closed conformation even in the absence of a bound ligand [53]. In our crystal structures, the α1 helix adopts a closed conformation, consistent with this gating mechanism, but with notable differences between the two ligands. **4** (4′-OPr) binds in a canonical orientation closely matching predicted docking poses and the established JH II/III binding mode, becoming fully buried within the protein core and reproducing the hydrophobic and polar contacts that stabilize the gate closed state. By contrast, **2** (4′-Bu) adopts an alternative orientation that opens the pocket outward on both sides to accommodate its hydrophobic alkylbenzyl moiety; this orientation perturbs the interaction network that normally stabilizes the fully closed conformation.

Gate closure normally promotes hydrogen bond formation between the N-terminal arm and the C-terminal tail, producing a fully gate closed JHBP–ligand complex [36]. In the open (apo) state, the α1 helix undergoes a large reorientation (≈70° toward the pocket) to open the pocket [36], whereas binding of **2** (4′-Bu) produces a smaller, qualitatively different shift that yields a partially open conformation. Consistent with this, **2** (4′-Bu) shows a larger hydrogen bond distance between nitrogen atom in imidazole moiety and Tyr128 residue and, also a comparatively lower binding affinity than JH II and **4** (4′-OPr). Moreover, as previously reported, researchers have identified the functional significance of interactions between hormones and proteins through structural investigations. They created 23 single mutants of JHBP, targeting 20 critical interacting residues, and assessed their JH-binding activity in vitro. As a result, the loss of binding activity observed in the Y128F mutant emphasizes the significant role of the hydrogen bond interaction between the OH group of Tyr128 and the epoxide oxygen of JH [36]. The structure of the JHBP–**4** complex displayed similar features, including a hydrogen-bonding interaction with the OH group in the Tyr128 residue and oxygen in the ether group in **4**, and is nearly identical to the conformations observed for JH II/III. The JHBP–**2** complex exhibits a similar interaction with the Tyr128 residue and nitrogen atom in imidazole group; however, it displays significant conformational differences compared to JH III and **4** (4′-OPr), probably due to the lack of an oxygen atom in the side chain. This distinct characteristic may indicate that **2** (4′-Bu) has weaker interactions with JHBP compared to **4** (4′-OPr). On the contrary, **2** (4′-Bu) may exert distinct biological effects through mechanisms in addition to—or distinct from—direct JHBP engagement, which could explain its association with acute toxicity. However, growth inhibition observed in the remaining unaffected individuals after the application of **2** (4′-Bu) may be attributed to JH antagonistic activity arising from interactions with JHBP.

Previous reports have linked acute lethality of certain MBIs bearing terpenoid side chains to respiratory inhibition via blockade of electron transfer between NADH and ubiquinone [27,49]. As an example, 1-(3,7-dimethyl-7-ethoxy-2-octenyl)-2-methylbenzimidazole (B-1) has demonstrated the strongest activity as a respiration inhibitor, exhibiting approximately six times the potency of rotenone. This compound has blocked the site between NADH and ubiquinone in the respiratory chain, indicating that the larvicidal benzimidazoles shared a mode of action with those of rotenone, piericidins, and ubicidines [49,54]. A similar mode of action is plausibly associated with the larvicidal activity of aliphatic alkyl-substituted BMBIs, such as compound **2** (4′-Bu), due to the fact that both the alkyl derivatives and B-1 share the common 2-methylbenzimidazole scaffold, despite substantial structural differences in the remaining moieties.

Conversely, the growth inhibitory and molting-related phenotypes induced by alkoxy derivatives are consistent with perturbations in chitin metabolism, indicating that the antagonistic activity of JH results from interactions with JHBP. Key enzymes in chitin turnover—chitinase, chitin synthase, and chitin deacetylase—are positively regulated by 2-hydroxyecdysone and tightly controlled by endocrine signaling; disruption of their activity or regulated expression impedes degradation of the old cuticle during ecdysis. For example, RNAi mediated suppression of Cht5 in *Bombyx mori* causes larval–larval molting failure [55]. Ecdysteroids and JH also regulate expression of cuticle associated genes, including cuticular protein analogous to peritrophins (CPAP) family members, which are essential for proper cuticle formation and development [56]. These regulatory links provide a mechanistic rationale for the molting defects observed with alkoxy substituted BMBIs. In summary, our combined biological, biophysical, and structural data indicate that subtle changes at the 4′-position of the benzyl ring can shift BMBI activity between acute toxicity and developmental disruption by altering ligand orientation, pocket dynamics, and possibly target engagement. These insights should inform rational design of next generation IGRs that maximize efficacy against pest species while minimizing off target toxicity to beneficial insects such as honeybees. Future work will focus on (i) direct assays of JH agonist/antagonist activity, (ii) evaluation of respiratory—chain inhibition for alkyl derivatives, and (iii) targeted studies of chitin metabolism and cuticle—related gene expression following exposure to representative alkoxy compounds.

## 5. Conclusions

In conclusion, the primary mode of action of MBIs appears to be disruption of JH signaling via inhibition of the JHBP, thereby perturbing the developmental regulation of precise JH titers. Ongoing studies will aim to achieve a more comprehensive understanding of binding interactions through molecular dynamics simulations.

## Figures and Tables

**Table 1:** Insecticidal activities (%) of 4′-alkyl and 4′-alkoxy BMBIs at 10 µg/larva against 3rd instar larvae.

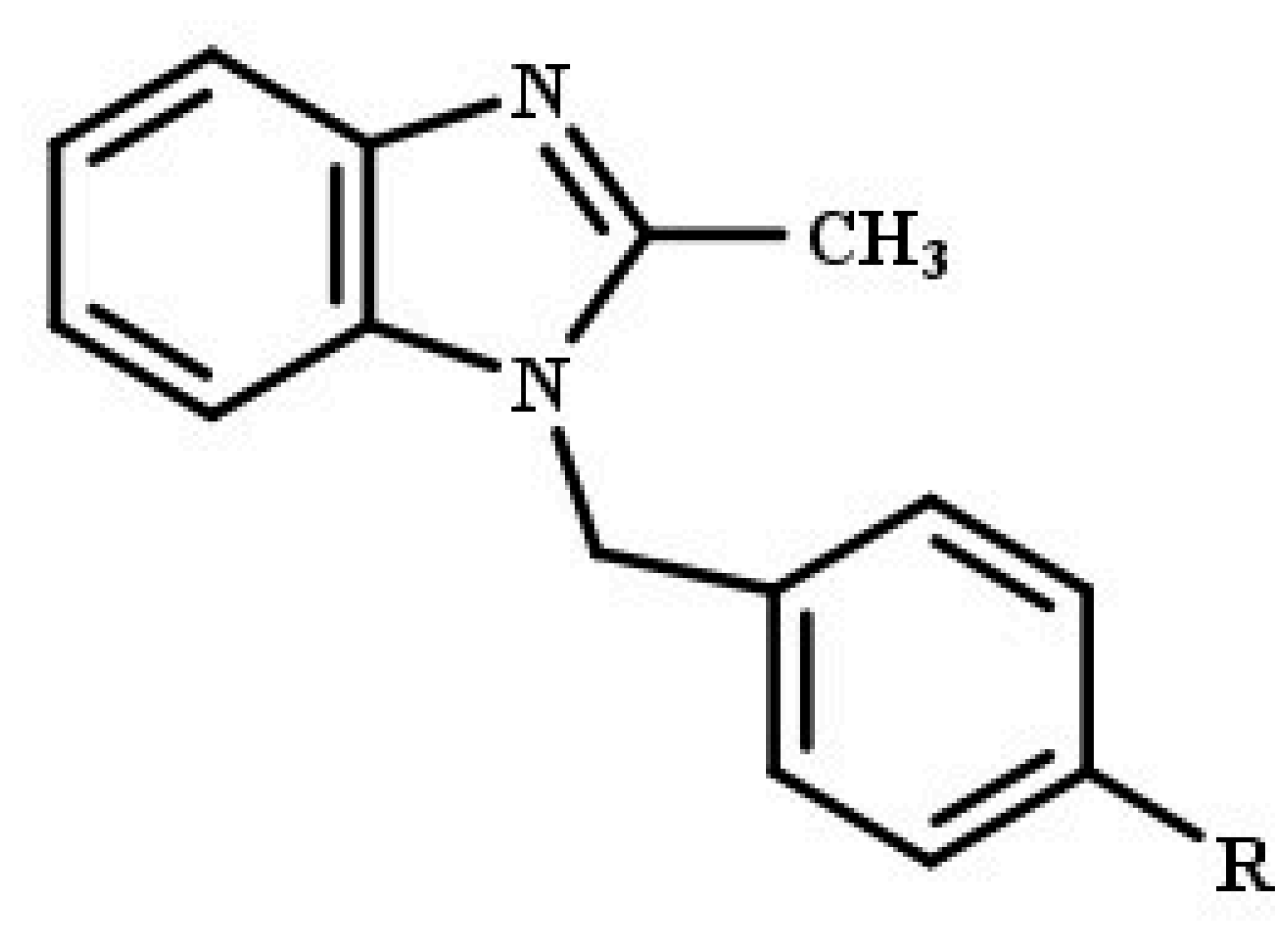	**Compounds**	**R Groups**	***N***	% of Biological Activity *	
				**Acute Toxicity**	**Growth Inhibition**
	**1**	4′-Pr	15	86.67 ± 11.55 ^a^	6.67 ± 11.55 ^c^
	**2**	4′-Bu	30	70.0 ± 32.86 ^a^	30.0 ± 32.86 ^abc^
	**3**	4′-Pe	15	93.33 ± 11.55 ^a^	6.67 ± 11.55 ^c^
	**4**	4′-OPr	20	0.0 ^b^	80.0 ± 16.33 ^a^
	**5**	4′-OBu	15	0.0 ^b^	20.0 ± 34.64 ^abc^
	**6**	4′-OPe	15	6.67 ± 11.55 ^b^	66.67 ± 11.55 ^ab^
	**7**	4′-OHx	15	0 ^b^	73.33 ± 11.55 ^a^
	Control		55	0 ^b^	14.44 ± 13.33 ^c^

* Data represents mean mortality rate (mean ± S.D.) after treatment with 10 µg/larva of BMBIs in day one 3rd instar silkworm larvae. Acetone was used as a control. Statistical significance was evaluated using one-way ANOVA followed by Games–Howell multiple comparisons test. Differences were considered statistically significant at *p* < 0.05. Different lowercase letters (a, b and c) indicate statistically significant differences among compounds (compounds sharing the same letter are not significantly different) based on the multiple comparison test, performed separately for acute toxicity rate and growth inhibition rate.

**Table 2:** Binding affinity of 4′-alkyl and 4′-alkoxy BMBIs with JHBP calculated using FREJIA.

Compounds	R Groups	EC_50_ (µM) ^a^	*n*
**1**	4′-Pr	2.75 ± 0.42	5
**2**	4′-Bu	1.22 ± 0.15	7
**3**	4′-Pe	1.63 ± 0.22	5
**4**	4′-OPr	2.08 ± 0.42	6
**5**	4′-OBu	2.10 ± 0.80	4
**6**	4′-OPe	1.65 ± 0.39	4
**7**	4′-OHx	1.07 ± 0.19	3
JH III		0.55 ± 0.08	6

^a^ Data represents mean half maximal effective concentration (EC50) ± S.D. Each assay was performed at least in triplicate.

**Table 3:** Molecular docking result of selected derivatives and JH II on the silkworm *Bombyx mori* JHBP (PDB: 3AOS).

Compounds	R Groups	Binding Affinity (kcal/mol)	Interacting Residue(s)
JH II		−8.4	^a^ (Tyr128), ^c^ (Phe24, Phe142), ^d^ (Val144, Ile208, Leu89, Leu25, Thr21, Ala20, Leu6, Leu17, Phe78), ^e^ (Met80, Val87, Tyr130, Ala212, Phe220, Phe221)
**4**	4′-OPr	−9.1	^a^ (Tyr128), ^b^ (Phe78) ^c^ (Phe220), ^d^ (Leu6, Phe24, Leu25, Val204, Thr91, Ile75, Leu17, Phe221, Val87), ^e^ (Leu89, Phe142, Ile208, Ala212, Val144), ^f^ (Thr21)
**2**	4′-Bu	−8.2	^a^ (Tyr128), ^b^ (Phe142, Phe220), ^d^ (Leu6, Ser211, Ile208, Leu89, Val87, Phe78, Met80, Thr21), ^e^ (Leu17, Ala20, Phe24, Leu215)

Superscripts a: Hydrogen bond, b: pi-pi T-shaped, c: pi -Sigma, d: van der Waals, e: Alkyl/pi-Alkyl Interaction, f: pi-Donor hydrogen bond.

## Data Availability

The data underlying this article are available in the article and on request. The coordinates and structure factors for the JHBP complexes with **2** (4′-OPr) and **4** (4′-Bu) have been deposited in the Protein Data Bank under accession codes 27GY and 27GZ, respectively.

## Supplementary Materials

The following supporting information can be downloaded at https://www.mdpi.com/article/10.3390/insects17060657/s1. Scheme S1. Synthetic route for the preparation of alkylamino MBI derivatives, Figure S1. Structures of synthesized compounds, Title. The synthetic procedures for the compounds used in the present study and characterization Data, Table S1 Data collection and structure solution statistics. Values for the outer shell are given in parentheses, Table S2. Insecticidal activities (%) of 4’-alkylamino BMBIs against day-1 3rd instar larvae, Figure S2. Biological activity of 4’-alkylamino BMBIs on day-1 3rd instar larvae at 10 µg/larva. The horizontal error bars denote S.D. Day-1 3rd instar B. mori larvae were topically treated with BMBIs dissolved in acetone. Acetone was used as control. Each assay was performed with a total of 15–55 larvae. Orange bars indicate acute mortality rate; Light green bars indicate growth inhibition rate. The x and y axes represent type of compound and mortality rate (%), respectively. Statistical significance was evaluated using one-way ANOVA followed by Games–Howell multiple comparisons test. Differences were considered statistically significant at *p* < 0.05, Figure S3. Survival curves after administration 10 µg/larva of 4’-alkylamino BMBIs to day-1 3rd instar larvae. Day-1 3rd instar B. mori larvae were topically treated with BMBIs dissolved in acetone, and survival rates were monitored for 30 days after treatment. Acetone was used as control. Each assay was performed with a total of 15–55 larvae. Data represents mean of survival rates. The x and y axes represent days after application and survival rate (%), respectively. The letters below the figure indicate the developmental stages of control larvae; M1 = moulting from 3rd to 4th instar, M2 = moulting from 4th to 5th, S = spinning, P = pupation. Colored areas in the figure denote durations of each instar; yellow = duration of 3rd instar, orange = duration of 4th instar, green = duration of 5th instar, and blue = pupal period, Table S3. Binding affinity of 4’-alkylamino BMBIs with JHBP calculated using FREJIA, Figure S4. Dose-response curve of 4’-alkylamino BMBIs on FRET activity. Data represents mean ± S.D. Each assay was performed at least in triplicate, Figure S5. Cartoon representation of the binding sites of three ligands within the JHBP, illustrating α helix conformational changes. (A) Binding interactions of JH II with JHBP; (B) Binding interactions of ligand 4′-OPr with JHBP; (C) Binding interactions of ligand 4′-Bu with JHBP.

## Abbreviations

The following abbreviations are used in this manuscript:

JH	Juvenile hormone
JHBP	Juvenile hormone binding protein
IGRs	Insect growth regulators
MBI	2-methylbenzimidazole
FRET	Förster resonance energy transfer
FREJIA	FRET JH indicator agent
