# Supplementary material for: Structural Characteristics for the Interaction of 1-Benzyl-2-Methylbenzimidazoles as Insect Growth Regulators and Juvenile Hormone Binding Protein
Source: Insects. 2026 Jun 22;17(6):657. doi: 10.3390/insects17060657 (PMC13300273; doi:10.3390/insects17060657)
Supplement: Supplementary file 1 [file insects-17-00657-s001.zip › [Paper no.2] Supplementary Materials_1_Synthetic Procedures and Characterization Data_20260614.pdf]

Submission to *Insects*

Mode of Action of 1-Benzyl-2-methylbenzimidazoles as Insect Growth Regulators through Interaction with Their Target Molecules

Udawaththa Kankanamge Don Sahan Suganda Gunasekara [1,†], Konatsu Inoue [2,†], Shuhei Henmi [2], Wataru Tsuchiya [3], Rintaro Suzuki [3], Keisuke Kutsuwada [3], Izumi Ikeda [4] and Takahiro Shiotsuki [1,2,4,*]

[1] *United Graduate School of Agricultural Sciences, Tottori University, Tottori, 680-0945, Japan.*

[2] *Graduate School of Natural Science and Technology, Shimane University, Matsue, 690-8504, Japan.*

[3] *Research Center for Advanced Analysis, National Agriculture and Food Research Organization, Tsukuba, Ibaraki 305-8642, Japan.*

[4] *Department of Life Science and Biotechnology, Faculty of Life and Environmental Science, Shimane University, Matsue, 690-8504, Japan.*

[*] Correspondence: shiotsuk@life.shimane-u.ac.jp

[†] These authors contributed equally to this work.

Supplementary Materials S1: Synthetic Procedures and Characterization Data

This file includes:

Scheme S1. Synthetic route

Figure S1. Structures of synthesized compounds.

Title. The synthetic procedures for the compounds used in the present study and characterization Data

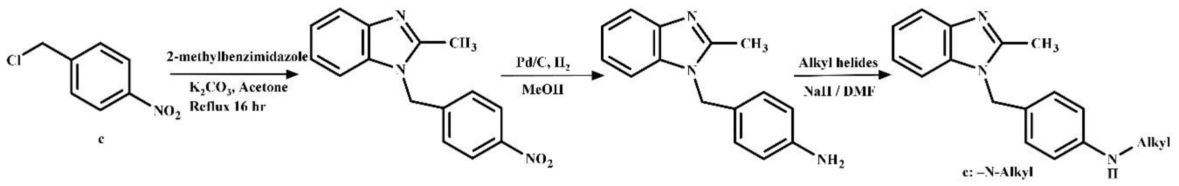

Scheme S1. Synthetic route for the preparation of alkylamino MBI derivatives

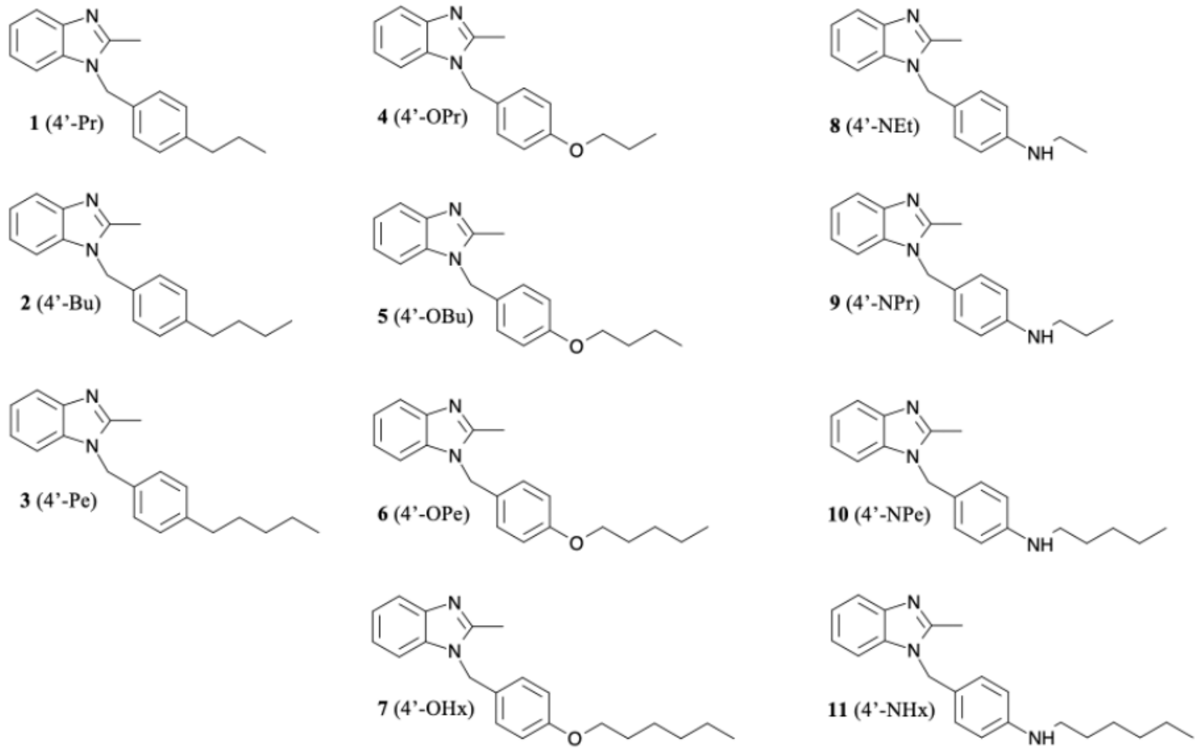

Figure S1. Structures of synthesized compounds

The synthetic procedures for the compounds used in the present study and characterization Data

2-Methyl-1-(4-propylbenzyl)-benzimidazole (**1** (4'-Pr))

4-Propylbenzoic acid (0.50 g, 3.1 mmol) was dissolved in THF (100 mL), followed by the sequential addition of $LiAlH_4$ (0.34 g, 8.96 mmol) suspended in THF (36 mL) at 0°C. The reaction mixture was then stirred under reflux at 66°C for 1 h and quenched with cooled distilled water and 10% aqueous NaOH (1.5 mL) to obtain 4-propylbenzyl alcohol as a colorless oil (0.51 g, 3.37 mmol, 91%).

The resulting 4-propylbenzyl alcohol (0.50 g, 3.34 mmol) in DCM (3mL) was treated with CBr_4 (1.1 g, 3.12 mmol) and PPh_3 (1.2 g, 4.15 mmol) in DCM (3mL) at 0°C, to afford 4-propylbenzyl bromide as a pale white oil (0.47 g, 2.22 mmol, 66%).

To obtain 2-methyl-1-(4-propylbenzyl)-benzimidazole, 4-propylbenzyl bromide (0.47 g, 2.21 mmol) was subjected to a S_N2 reaction in which 2-methylbenzimidazole (0.35 g, 2.67 mmol) in DMF (6 mL) was deprotonated with NaH (0.14 g, 2.91 mmol, 50% oil suspension) to afford the corresponding coupled product. The reaction mixture was purified by silica gel chromatography to obtain 2-methyl-1-(4-propylbenzyl)-benzimidazole (4'-Pr) as a yellow oil (0.37 g , 1.43 mmol, 54%); ^{1}H NMR δ_H (400 MHz, $CDCl_3$): 7.72 (d, J = 7.1 Hz , 1H), 7.26–7.18 (m, 3H), 7.11 (d, J = 8.0 Hz , 2H), 6.97 (d , 2H, J= 8.0 Hz), 5.29 (s, 2H), 2.57 (s, 3H), 2.54 (t, J = 7.7 Hz, 2H), 1.60 (q, J = 7.3 Hz , 2H), 0.91 (t, J =7.3 Hz, 3H); HRMS (ESI) m/z: calcd for $C_{18}H_{21}N_2$ $[M+H]^+$, 265.16993; Found, 265.1699.

1-(4-Butylbenzyl)-2-methylbenzimidazole (**2** (4'-Bu))

4-Butylbenzoic acid (0.50 g, 2.81 mmol) was dissolved in THF (10 mL), followed by the sequential addition of $LiAlH_4$ (1.0 g, 26.1 mmol) suspended in THF (36 mL) at 0°C. The reaction mixture was then stirred under reflux at 66°C for 1 h and quenched with cooled

distilled water and 10% aqueous NaOH (1.5 mL) to obtain 4-butylbenzyl alcohol as a colorless oil (0.47 g, 2.84 mmol, 98%).

The resulting 4-butylbenzyl alcohol (0.47 g, 2.86 mmol) in DCM (3mL) was treated with CBr_4 (1.06 g, 3.13 mmol) and PPh_3 (1.04 g, 3.98 mmol) in DCM (3mL) at 0°C, to afford 4-butylbenzyl bromide as a pale yellowish oil (0.22 g, 0.99 mmol, 59%).

To obtain 1-(4-butyllbenzyl)-2-methylbenzimidazole (4'-Bu), 4-butylbenzyl bromide (0.22 g, 0.97 mmol) was subjected to a S_N2 reaction in which 2-methylbenzimidazole (0.35 g, 2.67 mmol) in DMF (6 mL) was deprotonated with NaH (0.04 g, 0.83 mmol, 50% oil suspension) to afford the corresponding coupled product. The reaction mixture was purified by silica gel chromatography to obtain 1-(4-butyllbenzyl)-2-methylbenzimidazole (4'-Bu) as a pale yellow solid (0.15 g, 0.55 mmol, 72%); m.p. 46.1–46.8°C; ^{1}H NMR δ_H (400 MHz, $CDCl_3$): 7.72–7.71 (m, 1H), 7.25–7.18 (m, 3H), 7.10 (d, J = 8.0 Hz 2H), 6.96 (d, J = 8.0 Hz, 2H), 5.29 (s, 2H), 2.57 (s, 3H), 2.56 (t, J = 7.7 Hz, 2H), 1.64–1.51 (m, 2H), 1.37–1.26 (m, 2H), 0.63 (t, J = 7.4 Hz, 3H); HRMS (ESI) m/z: calcd for $C_{19}H_{23}N_2$ $[M+H]^+$, 279.18558; Found, 279.1856.

2-Methyl-1-(4-pentylbenzyl)-benzimidazole (**3** (4'-Pe))

4-Pentylbenzoic acid (0.51 g, 2.65 mmol) was dissolved in THF (10 mL), followed by the sequential addition of $LiAlH_4$ (0.30 g, 7.91 mmol) suspended with THF (36 mL) at 0°C. The reaction mixture was then stirred under reflux at 66°C for 1 h and quenched with cooled distilled water and 10% aqueous NaOH (1.5 mL) to obtain 4-pentylbenzyl alcohol as a colorless oil (0.44 g, 2.69 mmol, ca. 100%).

The resulting 4-pentylbenzyl alcohol (0.44 g, 2.69 mmol) in DCM (3mL) was treated with CBr_4 (1.07 g, 3.23 mmol) and PPh_3 (1.18 g, 4.51 mmol) in DCM (3mL) at 0°C, to afford 4-pentylbenzyl bromide as a pale yellowish oil (0.22 g, 0.91 mmol, 34%).

To obtain 2-methyl-1-(4-pentylbenzyl)-benzimidazole (4'-Pe), 4-pentylbenzyl bromide (0.22 g, 0.91 mmol) was subjected to a S_N2 reaction in which 2-methylbenzimidazole (0.15 g, 1.13 mmol) in DMF (6 mL) was deprotonated with NaH (0.07 g, 1.46 mmol, 50% oil suspension) to afford the corresponding coupled product. The reaction mixture was purified by silica gel chromatography to obtain 2-methyl-1-(4-pentylbenzyl)-benzimidazole (4'-Pe) as a pale yellow solid (0.223 g, 0.78 mmol, 86%); m.p. 45.0–47.3°C; ^{1}H NMR δ_H (400 MHz, $CDCl_3$): 7.73–7.70 (m, 1H), 7.28–7.18 (m, 3H), 7.11 (d, *J* = 7.8 Hz, 2H), 6.97 (d, *J* = 7.8 Hz, 2H), 5.29 (s, 2H), 2.57 (s, 3H), 2.56 (t, *J* = 7.7 Hz, 2H), 1.68–1.53 (m, 2H), 1.34–1.27 (m, 4H), 0.87 (t, *J* = 6.7 Hz, 3H); HRMS (ESI) m/z: calcd for $C_{20}H_{25}N_2$ $[M+H]^+$, 293.20123; Found, 293.2012.

1-(4-Butyloxybenzyl)-2-methylbenzimidazole (**5** (4'-OBu))

4-Hydroxybenzaldehyde (0.50 g, 4.09 mmol) was dissolved in DMF, followed by the sequential addition of K_2CO_3 (0.68 g, 4.93 mmol) and 1-bromobutane (0.67 g, 4.89 mmol). The mixture was then stirred to give 4-butoxybenzaldehyde (0.78 g, 4.38 mmol, quantitative).

The resulting 4-butoxybenzaldehyde (0.78 g, 4.38 mmol) was dissolved in MeOH (5 mL) and reduced to the corresponding benzyl alcohol by treatment with $NaBH_4$ (0.20 g, 5.29 mmol) under overnight stirring, affording 4-butoxybenzyl alcohol (0.81 g, 4.47 mmol, quantitative).

The 4-butoxybenzyl alcohol (0.81 g, 4.47 mmol) in DCM (10 mL) was treated with CCl_4 (0.43 mL, 4.42 mmol), and PPh_3 (1.2 g, 4.58 mmol), to give 4-butoxybenzyl chloride (0.17 g, 0.86 mmol, 19%).

To obtain 1-(4-butyloxybenzyl)-2-methylbenzimidazole (4'-OBu), 4-butoxybenzyl chloride (0.17 g, 0.86 mmol) was subjected to a S_N2 reaction in which 2-methylbenzimidazole (0.20 g, 1.51 mmol) in DMF (5 mL) was deprotonated with NaH (0.07 g, 1.46 mmol, 50% oil

suspension) to afford the corresponding coupled product. The reaction mixture was purified by silica gel chromatography to obtain 1-(4-butyloxybenzyl)-2-methylbenzimidazole (4'-OBu) as a yellowish solid (0.22 g, 0.73 mmol, 48%); m.p. 59.9–62.7°C; ^{1}H NMR δ_H (400 MHz, $CDCl_3$): 7.72–7.70 (m, 1H), 7.25–7.17 (m, 3H), 6.98 (d, J = 8.8 Hz, 2H), 6.85 (d, J = 8.5 Hz, 2H), 5.25 (s, 2H), 3.90 (t, J = 6.6 Hz, 2H), 2.57 (s, 3H), 1.76–1.71 (m, 2H), 1.49–1.42 (m, 2H), 0.95 (t, J = 7.3 Hz, 3H); HRMS (ESI) m/z: calcd for $C_{19}H_{23}ON_2$ $[M+H]^+$, 295.18049; Found, 295.1805.

2-Methyl-1-(4-pentyloxybenzyl)-benzimidazole (**6** (4'-OPe))

4-Hydroxybenzaldehyde (0.50 g, 4.09 mmol) was dissolved in DMF, followed by the sequential addition of K_2CO_3 (0.68 g, 4.93 mmol) and 1-bromopentane (0.74 g, 4.90 mmol). The mixture was then stirred to give 4-pentyloxybenzaldehyde (0.84 g, 4.36 mmol, quantitative).

The resulting 4-pentyloxybenzaldehyde (0.84 g, 4.36 mmol) was dissolved in MeOH (5 mL) and reduced to the corresponding benzyl alcohol by treatment with $NaBH_4$ (0.20 g, 5.29 mmol) under overnight stirring, affording 4-pentyloxybenzyl alcohol (0.84 g, 4.32 mmol, quantitative).

The 4-pentyloxybenzyl alcohol (0.84 g, 4.32 mmol) in DCM was treated with CCl_4 (0.67 g, 4.93 mmol), and PPh_3 (1.10 g, 4.19 mmol), to give 4-pentyloxybenzyl chloride (0.45 g, 2.12 mmol, 52%).

To obtain 2-methyl-1-(4-pentyloxybenzyl)-benzimidazole (4'-OPe), 4-pentyloxybenzyl chloride (0.45 g, 2.12 mmol) was subjected to a S_N2 reaction in which 2-methylbenzimidazole (0.34 g, 2.57 mmol) in DMF (5 mL) was deprotonated with NaH (0.12 g, 2.50 mmol, 50% oil suspension) to afford the corresponding coupled product. The reaction mixture was purified by silica gel chromatography to obtain 2-methyl-1-(4-pentyloxybenzyl)-benzimidazole (4'-OPe) as a white solid (86%, 0.69 g, 2.22 mmol); m.p. 61.6–63.5°C; ^{1}H NMR δ_H (400 MHz, $CDCl_3$): 7.72–7.70 (m, 1H), 7.25–7.17 (m, 3H), 6.97 (d, J = 4.4 Hz, 2H), 6.80

(d, J = 4.4 Hz, 2H), 5.25 (s, 2H), 3.90 (t, J = 6.6 Hz, 2H), 2.57 (s, 3H), 1.77–1.72 (m, 2H), 1.43–1.34 (m, 4H), 0.91 (t, J = 7.2 Hz, 3H); HRMS (ESI) m/z: calcd for $C_{20}H_{25}ON_2$ $[M+H]^+$, 309.19614; Found, 309.1961.

1-(4-Hexyloxybenzyl)-2-methylbenzimidazole (**7** (4'-OHx))

4-Hydroxybenzaldehyde (0.50 g, 4.09 mmol) was dissolved in DMF, followed by the sequential addition of K_2CO_3 (0.68 g, 4.93 mmol) and 1-bromohexane (0.81 g, 4.90 mmol). The mixture was then stirred to give 4-hexyloxybenzaldehyde (0.91 g, 4.41 mmol, 92%).

The resulting 4-hexyloxybenzaldehyde (0.91 g, 4.41 mmol) was dissolved in MeOH (5 mL) and reduced to the corresponding benzyl alcohol by treatment with $NaBH_4$ (0.20 g, 5.29 mmol) under overnight stirring, affording 4-hexyloxybenzyl alcohol (0.91 g, 4.36 mmol, 99%).

The 4-hexyloxybenzyl alcohol (0.91 g, 4.36 mmol) in DCM (10mL) was treated with CCl_4 (0.42 mL, 4.36 mmol), and PPh_3 (1.2 g, 4.58 mmol), affording 4-hexyloxybenzyl chloride (0.41 g, 1.81 mmol, 41%).

To obtain 1-(4-hexyloxybenzyl)-2-methylbenzimidazole (4'-OHx), 4-hexyloxybenzyl chloride (0.41 g, 1.81 mmol) was subjected to a S_N2 reaction in which 2-methylbenzimidazole (0.28 g, 2.12 mmol) in DMF (5 mL) was deprotonated with NaH (0.10 g, 2.08 mmol, 50% oil suspension), to afford the corresponding coupled product. The reaction mixture was purified by silica gel chromatography to obtain 1-(4-hexyloxybenzyl)-2-methylbenzimidazole (4'-OHx) as a pale yellow solid (0.58 g, 1.81 mmol, quantitative); m.p. 52.1–53.7°C; ^{1}H NMR δ_H (400 MHz, $CDCl_3$): 7.72–7.70 (m, 1H), 7.25–7.17 (m, 3H), 6.98 (d, J = 8.8 Hz, 2H), 6.91 (d, J = 8.8 Hz, 2H), 5.25 (s, 2H), 3.88 (t, J = 6.6 Hz, 2H), 2.55 (s, 3H), 1.77–1.70 (m, 4H), 1.33–1.24 (m, 4H), 0.89 (t, J = 5.6 Hz, 3H); HRMS (ESI) m/z: calcd for $C_{21}H_{27}ON_2$ $[M+H]^+$, 323.21179; Found, 323.2118.

1-(4-*N*-Ethylaminobenzyl)-2-methylbenzimidazole (**8** (4'-NEt))

2-Methylbenzimidazole (2.0 g, 15.0 mmol) was dissolved in acetone (150 mL), followed by the sequential addition of K_2CO_3 (2.5 g, 18.4 mmol) and 4-nitrobenzyl chloride (2.2 g, 12.8 mmol). The mixture was then stirred under reflux (60°C) for 16 h to obtain 1-(4-nitrobenzyl)-2-methylbenzimidazole as a pale yellow solid (1.6 g, 5.94 mmol, 49%).

The resulting 1-(4-nitrobenzyl)-2-methylbenzimidazole (0.84 g, 3.13 mmol) was dissolved in MeOH (25 mL). Subsequently, 10% Pd/C (0.02 g) was added, and the reaction mixture was stirred for 24 h to afford 1-(4-aminobenzyl)-2-methylbenzimidazole as a pale yellow powdery solid (0.71 g, 3.01 mmol, 96%).

To obtain 1-(4-*N*-ethylaminobenzyl)-2-methylbenzimidazole (4'-NEt), 1-(4-aminobenzyl)-2-methylbenzimidazole (0.11 g, 0.46 mmol) dissolved in DMF (4 mL) was treated with NaH (0.03 g, 0.63 mmol, 50% oil suspension) and iodoethane (0.23 g, 1.5 mmol). The reaction mixture was purified by silica gel chromatography to obtain 1-(4-*N*-ethylaminobenzyl)-2-methylbenzimidazole (4'-NEt) as a reddish brown sticky solid (0.02 g, 0.08 mmol, 18 %); m.p. 133.8–135.3°C; ^{1}H NMR δ_H (400 MHz, $CDCl_3$): 7.71 (d, J = 7.8 Hz, 1H), 7.29–7.20 (m, 3H), 6.90 (d, J = 8.2 Hz, 2H), 6.51 (d, J = 8.5 Hz, 2H), 5.21 (s, 2H), 3.11 (q, J =7.2 Hz, 2H), 2.60 (s, 3H), 1.23 (t, J =7.2 Hz, 3H)

2-Methyl-1-(4-*N*-propylaminobenzyl)-benzimidazole (**9** (4'-NPr))

2-Methylbenzimidazole (2.0 g, 15.0 mmol) was dissolved in acetone (150 mL), followed by the sequential addition of K_2CO_3 (2.5 g, 18.4 mmol) and 4-nitrobenzyl chloride (2.2 g, 12.8 mmol). The mixture was then stirred under reflux (60°C) for 16 h to obtain 1-(4-nitrobenzyl)-2-methylbenzimidazole as a pale yellow solid (1.6 g, 5.94 mmol, 49%).

The resulting 1-(4-nitrobenzyl)-2-methylbenzimidazole (0.84 g, 3.13 mmol) was dissolved in MeOH (25 mL). Subsequently, 10% Pd/C (0.02 g) was added, and the reaction

mixture was stirred for 24 h to afford 1-(4-aminobenzyl)-2-methylbenzimidazole as a pale yellow powdery solid (0.71 g, 3.01 mmol, 96%).

To obtain 2-methyl-1-(4-*N*-propylaminobenzyl)-benzimidazole (4'-NPr), 1-(4-aminobenzyl)-2-methylbenzimidazole (0.10 g, 0.42 mmol) dissolved in DMF (4 mL) was treated with NaH (0.03 g, 0.63 mmol, 50% oil suspension) and 1-bromopropane (0.253 g, 2.06 mmol). The reaction mixture was purified by silica gel chromatography to obtain 2-methyl-1-(4-N-propylaminobenzyl)-benzimidazole (4'-NPr) as a yellowish sticky solid (0.01 g, 0.04 mmol, 10%); m.p. 129.9–131.9°C; ^{1}H NMR δ_H (400 MHz, $CDCl_3$): 7.71 (d, *J* = 1.4 Hz, 1H), 7.69–7.17 (m, 3H), 6.90 (d, *J* = 8.5 Hz, 2H), 6.51 (d, *J* = 8.5 Hz, 2H), 5.20 (s, 2H), 3.04 (t, *J* =7.2 Hz, 2H), 2.58 (s, 3H), 1.61 (m, 2H), 0.97 (t, *J* =7.3 Hz, 3H)

2-Methyl-1-(4-*N*-pentylaminobenzyl)-benzimidazole (**10** (4'-NPe))

2-Methylbenzimidazole (2.0 g, 15.0 mmol) was dissolved in acetone (150 mL), followed by the sequential addition of K_2CO_3 (2.5 g, 18.4 mmol) and 4-nitrobenzyl chloride (2.2 g, 12.8 mmol). The mixture was then stirred under reflux (60°C) for 16 h to obtain 1-(4-nitrobenzyl)-2-methylbenzimidazole as a pale yellow solid (49%, 1.6 g, 5.94 mmol).

The resulting 1-(4-nitrobenzyl)-2-methylbenzimidazole (0.84 g, 3.13 mmol) was dissolved in MeOH (25 mL). Subsequently, 10% Pd/C (0.02 g) was added, and the reaction mixture was stirred for 24 h to afford 1-(4-aminobenzyl)-2-methylbenzimidazole as a pale yellow powdery solid (0.71 g, 3.01 mmol, 96%).

To obtain 2-methyl-1-(4-*N*-pentylaminobenzyl)-benzimidazole (4'-NPe), 1-(4-aminobenzyl)-2-methylbenzimidazole (0.08 g, 0.34 mmol) dissolved in DMF (4 mL) was treated with NaH (0.03 g, 0.63 mmol, 50% oil suspension) and 1-bromopentane (0.21 g, 1.32 mmol). The reaction mixture was purified by silica gel chromatography to obtain 2-methyl-1-(4-N-pentylaminobenzyl)-benzimidazole (4'-NPe) as a yellowish sticky solid (0.01 g, 0.03

mmol, 9%); m.p. 75.5–76.5°C; ^{1}H NMR δ_H (400 MHz, $CDCl_3$): 7.72 (d, *J* = 6.6 Hz, 1H), 7.71–7.17 (m, 3H), 6.90 (d, *J* = 8.5 Hz, 2H), 6.50 (d, *J* = 8.5 Hz, 2H), 5.20 (s, 2H), 3.05 (t, *J* =7.1 Hz, 2H), 2.58 (s, 3H), 1.48–1.32 (m, 6H), 0.90 (t, *J* =6.8 Hz, 3H)

1-(4-*N*-Hexylaminobenzyl)-2-methylbenzimidazole (**11** (4'-NHx))

2-Methylbenzimidazole (2.0 g, 15.0 mmol) was dissolved in acetone (150 mL), followed by the sequential addition of K_2CO_3 (2.5 g, 18.4 mmol) and 4-nitrobenzyl chloride (2.2 g, 12.8 mmol). The mixture was then stirred under reflux (60°C) for 16 h to obtain 1-(4-nitrobenzyl)-2-methylbenzimidazole as a pale yellow solid (1.6 g, 5.94 mmol, 49%).

The resulting 1-(4-nitrobenzyl)-2-methylbenzimidazole (0.84 g, 3.13 mmol) was dissolved in MeOH (25 mL). Subsequently, 10% Pd/C (0.02 g) was added, and the reaction mixture was stirred for 24 h to afford 1-(4-aminobenzyl)-2-methylbenzimidazole as a pale yellow powdery solid (0.71 g, 3.01 mmol, 96%).

To obtain 1-(4-*N*-hexylaminobenzyl)-2-methylbenzimidazole (4'-NHx), 1-(4-aminobenzyl)-2-methylbenzimidazole (0.12 g, 0.49 mmol) dissolved in DMF (4 mL) was treated with NaH (0.04 g, 0.73 mmol, 50% oil suspension) and 1-bromohexane (0.27 g, 1.64 mmol). The reaction mixture was purified by silica gel chromatography to obtain 1-(4-*N*-hexylaminobenzyl)-2-methylbenzimidazole (4'-NHx) as a yellowish sticky solid (0.01 g, 0.03 mmol, 6.1%); m.p. 89.4–93.6°C; ^{1}H NMR δ_H (400 MHz, $CDCl_3$): 7.71 (d, *J* = 1.5 Hz, 1H), 7.69–7.18 (m, 3H), 6.90 (d, *J* = 8.3 Hz, 2H), 6.50 (d, *J* = 8.3 Hz, 2H), 5.20 (s, 2H), 3.05 (t, *J* =7.2 Hz, 2H), 2.58 (s, 3H), 1.48–1.17 (m, 8H), 0.90 (t, *J* =6.8 Hz, 3H)
