# Supplementary material for: Structural Characteristics for the Interaction of 1-Benzyl-2-Methylbenzimidazoles as Insect Growth Regulators and Juvenile Hormone Binding Protein
Source: Insects. 2026 Jun 22;17(6):657. doi: 10.3390/insects17060657 (PMC13300273; doi:10.3390/insects17060657)
Supplement: Supplementary file 1 [file insects-17-00657-s001.zip › [Paper no.2] Supplementary Materials_2_Data on alkylamino BMBIs_20260614.pdf]

Submission to *Insects*

Mode of Action of 1-Benzyl-2-methylbenzimidazoles as Insect Growth Regulators through Interaction with Their Target Molecules

Udawaththa Kankanamge Don Sahan Suganda Gunasekara [1,†], Konatsu Inoue [2,†], Shuhei Henmi [2], Wataru Tsuchiya [3], Rintaro Suzuki [3], Keisuke Kutsuwada [3], Izumi Ikeda [4] and Takahiro Shiotsuki [1,2,4,*]

[1] *United Graduate School of Agricultural Sciences, Tottori University, Tottori, 680-0945, Japan.*

[2] *Graduate School of Natural Science and Technology, Shimane University, Matsue, 690-8504, Japan.*

[3] *Research Center for Advanced Analysis, National Agriculture and Food Research Organization, Tsukuba, Ibaraki 305-8642, Japan.*

[4] *Department of Life Science and Biotechnology, Faculty of Life and Environmental Science, Shimane University, Matsue, 690-8504, Japan.*

[*] Correspondence: shiotsuk@life.shimane-u.ac.jp

[†] These authors contributed equally to this work.

Supplementary Materials S2: Biological Activity Data

This file includes:

Table S1 Data collection and structure solution statistics. Values for the outer shell are given in parentheses.

Table S2. Insecticidal activities (%) of 4'-alkylamino BMBIs against day-1 3rd instar larvae.

Figure S2. Biological activity of 4'-alkylamino BMBIs on day-1 3rd instar larvae at 10 μg/larva. The horizontal error bars denote S.D. Day-1 3rd instar B. mori larvae were topically treated with BMBIs dissolved in acetone. Acetone was used as control. Each assay was performed with a total of 15–55 larvae. Orange bars indicate acute mortality rate; Light green bars indicate growth inhibition rate. The x and y axes represent type of compound and mortality rate (%), respectively. Statistical significance was evaluated using one-way ANOVA followed by Games–Howell multiple comparisons test. Differences were considered statistically significant at $p < 0.05$.

Figure S3. Survival curves after administration 10 μg/larva of 4'-alkylamino BMBIs to day-1 3rd instar larvae. Day-1 3rd instar B. mori larvae were topically treated with BMBIs dissolved

in acetone, and survival rates were monitored for 30 days after treatment. Acetone was used as control. Each assay was performed with a total of 15–55 larvae. Data represents mean of survival rates. The x and y axes represent days after application and survival rate (%), respectively. The letters below the figure indicate the developmental stages of control larvae; M1 = moulting from 3rd to 4th instar, M2 = moulting from 4th to 5th, S = spinning, P = pupation. Colored areas in the figure denote durations of each instar; yellow = duration of 3rd instar, orange = duration of 4th instar, green = duration of 5th instar, and blue = pupal period.

Table S3. Binding affinity of 4'-alkylamino BMBIs with JHBP calculated using FREJIA.

Figure S4. Dose-response curve of 4'-alkylamino BMBIs on FRET activity. Data represents mean ± S.D. Each assay was performed at least in triplicate.

Figure S5. Cartoon representation of the binding sites of three ligands within the JHBP, illustrating α helix conformational changes. (A) Binding interactions of JH II with JHBP; (B) Binding interactions of ligand 4'-OPr with JHBP; (C) Binding interactions of ligand 4'-Bu with JHBP.

Table S1 Data collection and structure solution statistics. Values for the outer shell are given in parentheses.

Structure (PDB ID)	JHBP–4′-OPr (27GY)	JHBP–4'-Bu (27GZ)
Data collection		
Diffraction source	Photon Factory BL-5A	Photon Factory AR-NW12A
Wavelength (Å)	1.0000	1.0000
Temperature (K)	95	95
Detector	PILATUS 2M	PILATUS 2M
Total rotation range (°)	360	360
Space group	$R32$	$P6_322$
a, b, c (Å)	127.7, 127.7, 156.7	88.1, 88.1, 135.5
α, β, γ (°)	90, 90, 120	90, 90, 120
Mosaicity (°)	0.57	0.51
Resolution range (Å)	100–1.85 (1.92–1.85)	100–2.40 (2.53–2.40)
Total No. of reflections	822 643	472 097
No. of unique reflections	41 938 (4 145)	12 778 (1 812)
Completeness (%)	100.0 (100.0)	100.0 (100.0)
Redundancy	19.6 (18.4)	36.9 (38.4)
$\langle I/\sigma(I) \rangle$	44.5 (2.8)	19.3 (2.3)
R-factor	0.031 (0.669)	0.180 (0.951)
$R_{p.i.m.}$	0.011 (0.198)	0.029 (0.333)
Structure solution		
Resolution range (Å)	36.96–1.85 (1.90–1.85)	38.89–2.40 (2.45–2.40)
No. of reflections	39 860 (3043)	12 054 (916)
Final R_{work}	0.186 (0.263)	0.235 (0.320)
Final R_{free}	0.207 (0.301)	0.283 (0.385)
RMS deviations from ideal		
Bonds (Å)	0.006	0.003
Angles (°)	1.300	0.941
Average B factors (Å^2)	34.4	58.3
Ramachandran plot		
Most favoured (%)	98.2	96.8
Allowed (%)	1.8	3.2
Disallowed (%)	0.0	0.0

Table S2. Insecticidal activities (%) of 4’-alkylamino BMBIs against day-1 3rd instar larvae.

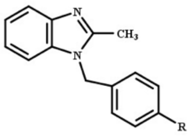

Compounds	R groups	N	% of biological activity*	
			Acute toxicity	Growth inhibition
**8**	4'-NEt	15	0^{b}	6.67 ± 11.55^{c}
**9**	4'-NPr	15	0^{b}	13.33 ± 11.55^{bc}
**10**	4'-NPe	15	0^{b}	13.33 ± 11.55^{bc}
**11**	4'-NHx	15	0^{b}	33.33 ± 11.55^{abc}
Control		55	0^{b}	14.44 ± 13.33^{c}

*Mean mortality rate (mean ±S.D) after treatment with 10 μg/larva of BMBIs in day-1 3rd instar silkworm larvae. Acetone was used as a control. Statistical significance was evaluated using one-way ANOVA followed by Games–Howell multiple comparisons test. Differences were considered statistically significant at $p < 0.05$. Different lowercase letters (a, b and c) indicate statistically significant differences among compounds (compounds sharing the same letter are not significantly different) based on the multiple comparison test, performed separately for acute toxicity rate and growth inhibition rate.

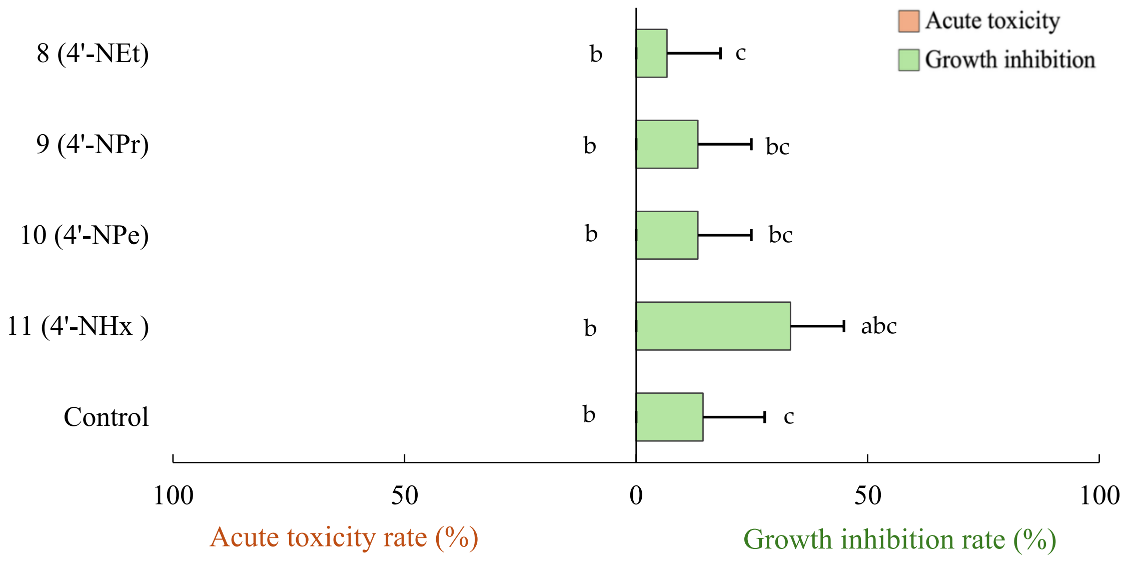

Figure S2. Biological activity of 4'-alkylamino BMBIs on day-1 3rd instar larvae at 10 μg/larva. The horizontal error bars denote S.D. Day-1 3rd instar B. mori larvae were topically treated with BMBIs dissolved in acetone. Acetone was used as control. Each assay was performed with a total of 15–55 larvae. Orange bars indicate acute mortality rate; Light green bars indicate growth inhibition rate. The x and y axes represent type of compound and mortality rate (%), respectively. Statistical significance was evaluated using one-way ANOVA followed by Games–Howell multiple comparisons test. Differences were considered statistically significant at $p < 0.05$. Different lowercase letters (a, b and c) indicate statistically significant differences among compounds (compounds sharing the same letter are not significantly different) based on the multiple comparison test, performed separately for acute toxicity rate and growth inhibition rate.

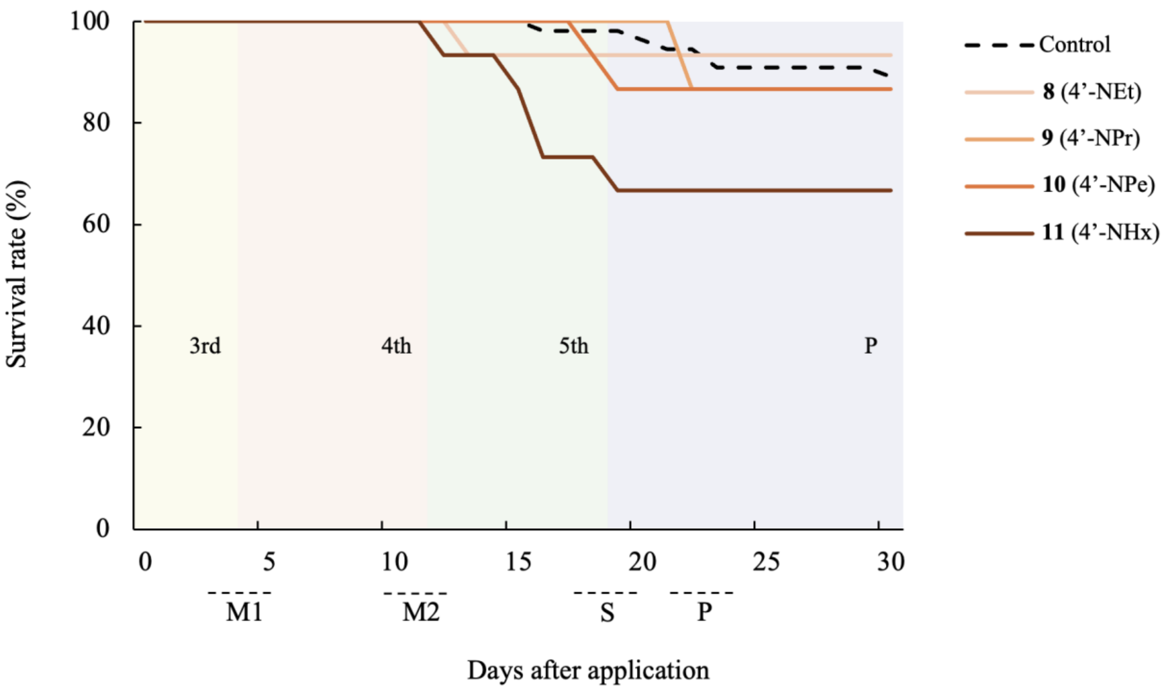

Figure S3. Survival curves after administration 10 µg/larva of 4’-alkylamino BMBIs to day-1 3rd instar larvae. Day-1 3rd instar *B. mori* larvae were topically treated with BMBIs dissolved in acetone, and survival rates were monitored for 30 days after treatment. Acetone was used as control. Each assay was performed with a total of 15–55 larvae. Data represent mean survival rates. The x and y axes represent days after application and survival rate (%), respectively. The letters below the figure indicate the developmental stages of control larvae; M1 = moulting from 3rd to 4th instar, M2 = moulting from 4th to 5th, S = spinning, P = pupation. Colored areas in the figure denote durations of each instar; yellow = duration of 3rd instar, orange = duration of 4th instar, green = duration of 5th instar, and blue = pupal period.

Table S3. Binding affinity of 4'-alkylamino BMBIs with JHBP calculated using FREJIA.

Compounds	R groups	EC_{50} (μM)[a]	*n*
**8**	4-NEt	10.09 ± 1.96	3
**9**	4-NPr	6.79 ± 3.06	6
**10**	4-NPe	4.64 ± 1.38	3
**11**	4-NHx	2.14 ± 0.21	3
JH III		0.55 ± 0.08	6

[a]Data represents mean half maximal effective concentration (EC_{50}) ± S.D. Each assay was performed at least in triplicate.

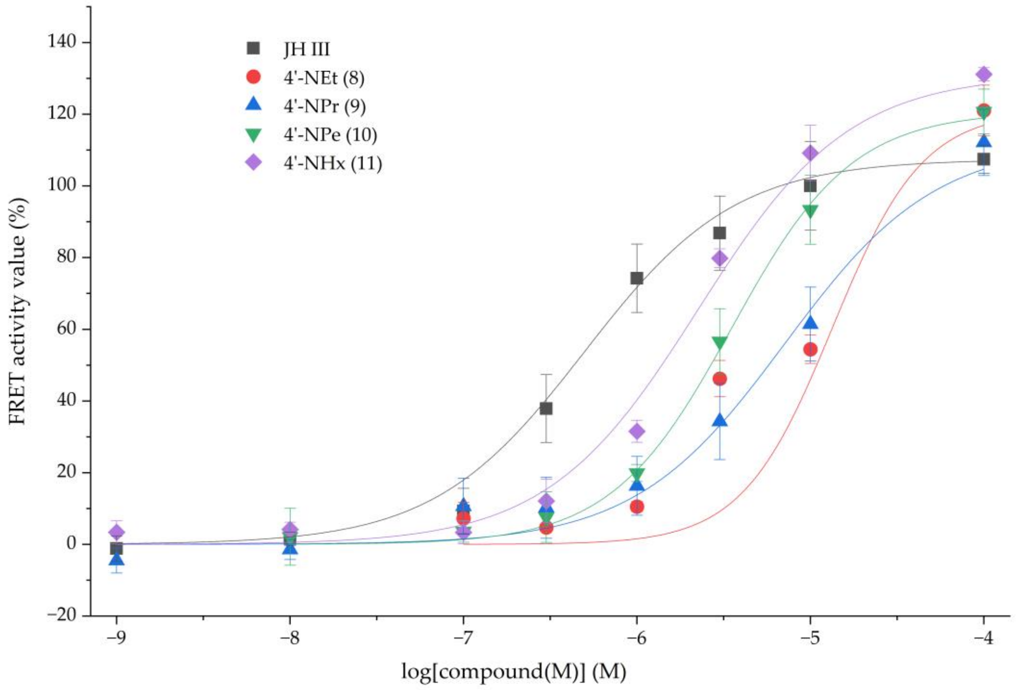

Figure S4. Dose-response curve of 4'-alkylamino BMBIs on FRET activity. Data represents mean±S.D. Each assay was performed at least in triplicate.

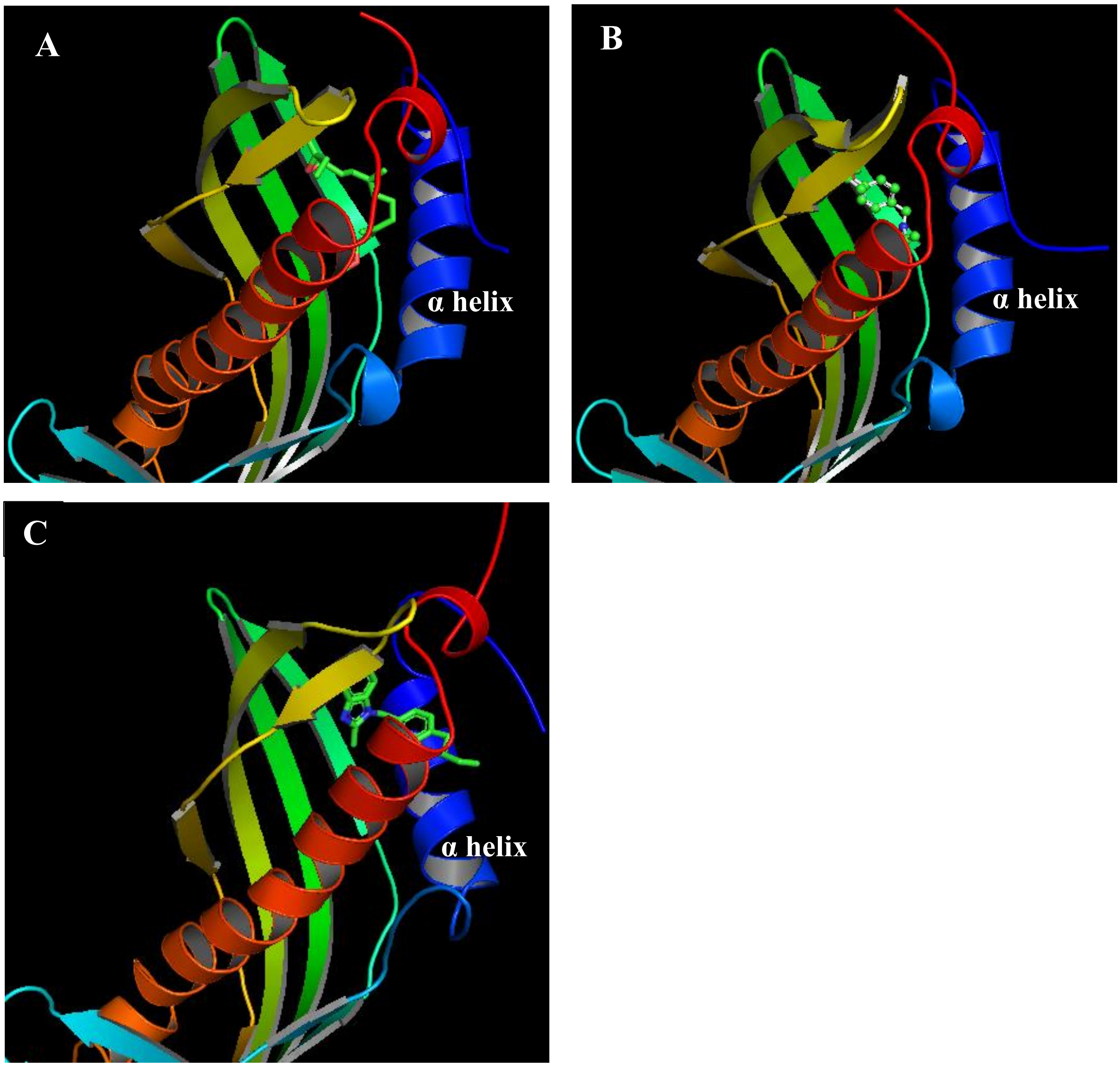

Figure S5. Cartoon representation of the binding sites of three ligands within the JHBP, illustrating α helix conformational changes. (A) Binding interactions of JH II with JHBP; (B) Binding interactions of ligand 4'-OPr with JHBP; (C) Binding interactions of ligand 4'-Bu with JHBP.
